# The multidimensional nature of aphasia recovery post-stroke

**DOI:** 10.1093/brain/awab377

**Published:** 2022-03-10

**Authors:** James D Stefaniak, Fatemeh Geranmayeh, Matthew A Lambon Ralph

**Affiliations:** 1 MRC Cognition and Brain Sciences Unit, University of Cambridge, Cambridge CB2 7EF, UK; 2 Department of Psychiatry, University of Cambridge, Cambridge CB2 0SZ, UK; 3 Division of Neuroscience and Experimental Psychology, School of Biological Sciences, University of Manchester, Manchester Academic Health Science Centre, Manchester M13 9GB, UK; 4 Computational Cognitive and Clinical Neuroimaging Laboratory, Department of Brain Sciences, Imperial College London, Hammersmith Hospital Campus, London W12 0NN, UK

**Keywords:** aphasia, stroke, plasticity, fMRI

## Abstract

Language is not a single function, but instead results from interactions between neural representations and computations that can be damaged independently of each other. Although there is now clear evidence that the language profile in post-stroke aphasia reflects graded variations along multiple underlying dimensions (‘components’), it is still entirely unknown if these distinct language components have different recovery trajectories and rely on the same, or different, neural regions during aphasia recovery. Accordingly, this study examined whether language components in the subacute stage: (i) mirror those observed in the chronic stage; (ii) recover together in a homogeneous manner; and (iii) have recovery trajectories that relate to changing activation in distinct or overlapping underlying brain regions.

We analysed longitudinal data from 26 individuals with mild–moderate aphasia following left hemispheric infarct who underwent functional MRI and behavioural testing at ∼2 weeks and ∼4 months post-stroke. The language profiles in early post-stroke aphasia reflected three orthogonal principal components consisting of fluency, semantic/executive function and phonology. These components did not recover in a singular, homogeneous manner; rather, their longitudinal trajectories were uncorrelated, suggesting that aphasia recovery is heterogeneous and multidimensional. Mean regional brain activation during overt speech production in unlesioned areas was compared with patient scores on the three principal components of language at both the early and late time points. In addition, the change in brain activation over time was compared with the change on each of the principal component scores, both before and after controlling for baseline scores. We found that different language components were associated with changing activation in multiple, non-overlapping bilateral brain regions during aphasia recovery. Specifically, fluency recovery was associated with increasing activation in bilateral middle frontal gyri and right temporo-occipital middle temporal gyrus; semantic/executive recovery was associated with reducing activation in bilateral anterior temporal lobes; while phonology recovery was associated with reducing activation in bilateral precentral gyri, dorso-medial frontal poles and the precuneus. Overlapping clusters in the ventromedial prefrontal cortex were positively associated with fluency recovery but negatively associated with semantic/executive and phonology recovery.

This combination of detailed behavioural and functional MRI data provides novel insights into the neural basis of aphasia recovery. Because different aspects of language seem to rely on different neural regions for recovery, treatment strategies that target the same neural region in all stroke survivors with aphasia might be entirely ineffective or even impair recovery, depending on the specific language profile of each individual patient.

## Teaser

Stefaniak *et al*. report that (i) the language profile is multidimensional in subacute post-stroke aphasia; (ii) aphasia recovery is heterogeneous and does not reflect a single, unitary process; and (iii) recovery of different language dimensions is related to changes in task-induced activation in different brain regions.

## Introduction

Aphasia affects at least one-third of the more than 10 million new stroke cases globally each year.^1,2^ Despite many stroke survivors exhibiting spontaneous partial language recovery,^3^ difficulties persist into the chronic phase in at least 40% of initially aphasic patients.^4^ There is, therefore, an urgent need to understand the mechanisms involved in language recovery so that we can develop targeted treatments for this costly and debilitating condition.^5-7^

Functional neuroimaging can provide empirical evidence regarding the patterns of neural activity that are associated with language performance post-stroke,^8–12^ which in turn might help to adjudicate between which underlying recovery mechanisms occur *in vivo*.^5^ Language recovery occurs most rapidly during the first few months post-stroke,^13^ suggesting that imaging correlates of language performance in the subacute phase would provide the most information regarding recovery mechanisms. Despite the clear need for investigation of this early period post-stroke, a recent formal meta-analysis of the functional MRI aphasia literature found that studies were dominated by examination of very chronic cases (the median time post-onset across the literature was 38 months) and only a handful of studies provided longitudinal data albeit often in small samples (typical *n* < 10) with minimal behavioural data,^14^ crucial for understanding how behavioural variation relates to the functional MRI results. The relative lack of studies, small sample sizes and minimal behavioural data are unsurprising given the considerable logistic challenges involved in recruiting, assessing and scanning subacute stroke patients. However, longitudinal studies are a powerful approach for exploring the neural bases of recovery (because the different starting points and inter-participant variations are controlled) and, particularly, for exploring whether language network changes observed in the chronic phase occur immediately or over time. Accordingly, this study utilized detailed behavioural data and task-based functional MRI in a larger post-stroke aphasia group at both early (2 weeks) and later (4 months) time points.

Among the handful of acute-to-chronic longitudinal post-stroke aphasia studies, several have associated better language recovery with increasing activation in regions of both hemispheres that are known to be part of the premorbid language network, including the left superior temporal gyrus (STG),^15,16^ left posterior temporal lobe,^12,17^ left inferior frontal gyrus (IFG),^12^ right anterior temporal lobe (ATL),^12^ right STG,^15,18^ right posterior temporal lobe^17^ and right IFG.^19^ Investigations have also associated better language recovery with increasing activation in domain-general executive regions,^20^ including the medial prefrontal cortex,^21,22^ left IFG pars opercularis and anterior insula,^12^ left dorsolateral prefrontal cortex,^18^ right anterior insula^21^ and right dorsolateral prefrontal cortex.^12,18^ Regions associated with recovery in these studies were often activated in controls performing the same language task,^12,15,18,19,21^ consistent with the variable neuro-displacement principle, in which recovery follows from upregulation of the dynamic, spare capacity found in both undamaged regions of premorbid language networks and domain-general executive regions during subacute aphasia recovery.^5^

As noted above, a second major limitation of previous longitudinal studies of subacute post-stroke aphasia is the limited variety and depth of behavioural data. Typically, neuroimaging data are either correlated with a single behavioural score^12,15,16,21-23^ or with a few language scores that cover a limited segment of the full language profile.^17–19^ However, language is not a single, homogeneous cognitive function but is instead subserved by interactions between more general cognitive computations that can be damaged independently of each other,^24,25^ thereby contributing to the multidimensional profile of chronic post-stroke aphasia.^26–30^ In a cross-sectional study, Kümmerer *et al*.^31^ used a data-driven decomposition technique, principal component analysis (PCA), to investigate the existence of distinct domains of language performance in subacute post-stroke aphasia and identified two components, representing ‘comprehension’ and ‘repetition’, that were associated with the degree of damage to the ventral and dorsal language pathways, respectively. Although there is now clear evidence that both subacute and chronic post-stroke aphasia reflects graded multidimensional variations, to date longitudinal studies have not investigated whether these distinct underlying components of language have different recovery trajectories, and if so, whether they are associated with changing activation in different or overlapping neural regions. Accordingly, this was a key target for the present study.

This study analysed data from one of the largest longitudinal functional neuroimaging studies in subacute post-stroke aphasia to date.^22^ The first question we addressed was whether the subacute language profile can be dissociated into orthogonal principal components that mirror those observed in the chronic stage, i.e. phonology,^26^ semantics,^26^ fluency^28^ and executive function.^29^ The second question was whether such distinct underlying components recover in a homogeneous manner, which we would expect if language recovery occurred uniformly along a single dimension, or whether the distinct behavioural components recover in a manner that is uncorrelated, which would suggest that aphasia recovery is multidimensional. The third question was whether different language components are associated with changing activation in distinct or overlapping neural regions during aphasia recovery. This has significant clinical implications; if different language components rely on different neural regions for recovery, then targeting the same neural region in all aphasic stroke survivors might be ineffective or even impair recovery, depending on the specific language profile of each individual patient.

In this study, we also examined a potentially crucial methodological issue for the first time. Perhaps unsurprisingly and unavoidably given the significant clinical challenges associated with the phase immediately after a stroke, the handful of studies that have examined acute post-stroke aphasia have recruited patients with mild–moderate aphasia. However, patients with mild aphasia in the subacute phase tend to recover well, resulting in their data at the chronic time point approaching ceiling and having reduced variance.^12,15,18,22^ This change in the shape of the data over time has important statistical consequences when examining the behavioural or imaging correlates of performance change. Recent work on the ‘proportional recovery rule’ has demonstrated that, when chronic scores are less variable than at baseline, baseline scores inevitably become negatively correlated with subsequent performance change, even in the complete absence of any relationship between baseline and chronic scores.^32^ To our knowledge, no existing longitudinal imaging study has adequately accounted for baseline language performance during whole-brain image analysis. Consequently, regions identified during whole-brain image analysis in which activation change was associated with language recovery could be ‘false positives’, confounded by a true association between activation and baseline performance that is sufficient to account for the observed association with recovery. Additionally, there could be ‘false negatives’, in which a true association between activation and language recovery has been masked by varying baseline performance. This issue was examined formally in the current study.

## Materials and methods

### Participants

Twenty-seven individuals with a left hemisphere infarct and language difficulties at stroke onset underwent a battery of language assessments and MRI scans at 2 weeks (time point 1, T1) and 4 months (time point 2, T2) post-stroke. Patients were premorbidly fluent in English and did not have a previous history of stroke resulting in aphasia or other neurological condition. Twenty-six patients had a full battery of language test data available and were included in this analysis. Demographic and clinical variables for the post-stroke aphasia group are shown in Supplementary Table 1. Twenty-four right-handed, fluent English-speaking controls without history of neurological impairment were recruited for comparison, 22 of whom underwent two MRI scans over the same inter-scan interval as the patients. The 26 patients and 22 controls were age-matched [mean 59.0 (standard deviation, SD 11.0) years in post-stroke aphasia versus 57.6 (SD 10.7) in controls; *t*-test, *t*(46) = 0.45, *P* = 0.66]. Informed consent was obtained from all participants according to the Declaration of Helsinki under approval by the National Research Ethics Service Committee. These data were used for previous publications which focused on neuroimaging correlates for a single language measure.^8,22,33,34^ The current study provides entirely new results from extensive, novel analyses of the entire, combined behavioural and neuroimaging data spanning the two time points. In doing so, we were able to address the key study questions set out in the Introduction.

### Neuropsychological tests

At both T1 and T2, patients completed subsections of the Comprehensive Aphasia Test^35^ yielding scores on ‘Spoken Picture Description’, ‘Fluency’, ‘Spoken Comprehension’, ‘Written Comprehension’, ‘Repetition’, ‘Object Naming’, ‘Reading’ and ‘Cognitive’. They also underwent a brief version of the Raven’s Progressive Matrix Test^36^ and a quantitative analysis of connected speech production based on their narrative recollection of the Cinderella story yielding ‘Information Carrying Words (ICWs) per second’, ‘Syllables per second’, ‘total ICWs’, and a ‘Narrative Aphasia Score’ (NAS; see Supplementary material).^37,38^ In addition, several behavioural measures were obtained from their performance during the functional MRI scan, including: Inverse Efficiency Score (mean reaction time in seconds/proportion correct) from the ‘Decision’ task (see below for task details); ‘Appropriate ICWs minus Inappropriate ICWs’^35^ from the ‘Speech’ task (see below for task details); and ‘Syllable Rate’ from the ‘Speech’ task. This yielded 16 neuropsychological measures per patient at both T1 and T2. A subset of these neuropsychological measures was available in the controls at both time points. Neuropsychological test scores are shown in Supplementary Tables 2 and 3.

### Statistical analysis

We used varimax-rotated PCA to reduce the patients’ scores across all 16 neuropsychological tests, to a smaller number of principal components (PCs), taken to be estimates of underlying cognitive dimensions. This data-driven decomposition technique has previously demonstrated the existence of orthogonal cognitive domains underlying observed variation in neuropsychological performance in chronic post-stroke aphasia.^26–28,39^ Varimax-rotated PCA was performed on the 16 neuropsychological scores of patients, separately at T1 and T2. We used SPSS version 25 for the above statistical analyses. *P*-values are two-tailed and reported in their uncorrected form; alpha was set at 0.05, with statistical significance determined after applying a Bonferroni correction to the threshold (i.e. dividing 0.05 by the number of comparisons).

### Lesion overlap map

**Figure 1:**
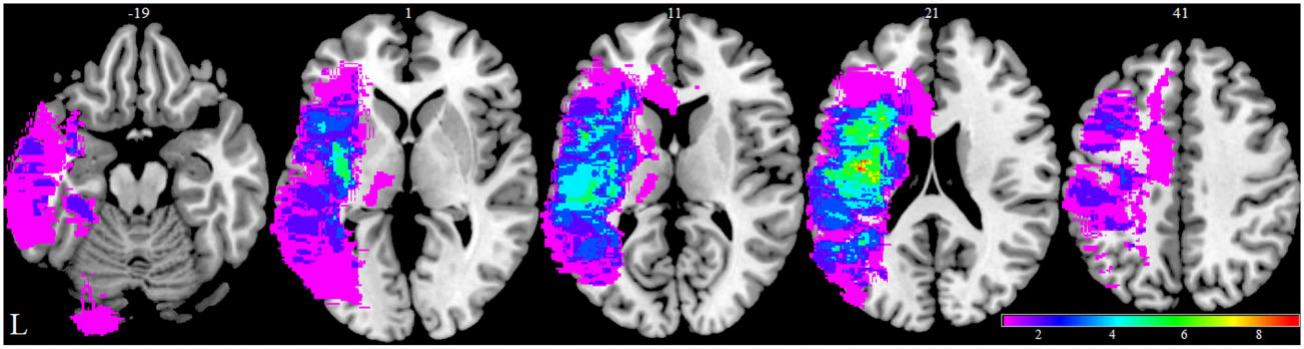
**Lesion overlap map.** Lesion overlap map for the 26 patient post-stroke aphasia subgroup in Montreal Neurological Institute (MNI) space and in neurological convention using time point 1 scans (2 weeks post-stroke). Colour bar represents overlap number between 1 and 9. The numbers refer to the MNI coordinate space in the *z* plane. L = left.

The lesion overlap map of the post-stroke aphasia subgroup is shown in Fig. 1; it encompasses most of the left hemisphere, middle cerebral artery territory including subcortical white matter.^40,41^

### Functional MRI design

For details of MRI data acquisition see Supplementary material. A sparse functional MRI design was used to minimize motion artefact by acquiring echo-planar imaging (EPI) volumes in between periods of speech production.^42,43^ Each scanning session contained three functional MRI runs. Each run contained 20 ‘Speech’ trials, 16 ‘Count’ trials, 16 ‘Decision’ trials and 15 ‘Rest’ trials. Each ‘Speech’ trial required participants to overtly define an object shown as a coloured picture; the speech produced was recorded with an Optacoustics FOMRI-III microphone, transcribed and analysed to yield the behavioural measures described previously. ‘Count’ trials required participants to count from one to seven at a rate of one per second; ‘Decision’ trials required participants to press a button when presented with a blue square but inhibit their response when presented with an orange circle; and ‘Rest’ trials required participants to view a fixation cross. Trials were presented in blocks of two or four of the same type, with each of the four ‘Decision’ blocks per run being preceded by a 10 s instruction page. Each trial lasted 10 s; the task was performed during the first 7 s before being terminated by a fixation cross, with the EPI functional MRI volume being acquired 1 s later over 2 s. Thus, one EPI volume was acquired per trial, and 71 volumes were acquired over each run.

### Functional MRI first-level analysis

Each subject’s EPI volumes were realigned, brain-extracted, smoothed, coregistered and normalized using FMRIB Software Library (www.fmrib.ox.ac.uk/fsl)^44–46^ and Statistical Parametric Mapping (SPM) 12 (Wellcome Centre for Human Neuroimaging, London UK; www.fil.ion.ucl.ac.uk/spm/).^47^ See the Supplementary material for more detail. The preprocessed data were then entered into a fixed-effect first-level analysis using the general linear model in which each 10 s trial was modelled as an epoch convolved with the canonical haemodynamic response function.^48^ Regressors were resampled 9 s into each 10-s trial, which was the half-way point of each EPI volume’s acquisition in this sparse scanning design. Six rigid-body motion parameters were included as regressors of no-interest. Each subject’s three runs were modelled as separate sessions in the same first-level design matrix.

The contrast of interest was the summation of ‘Speech and Count’ over ‘Rest’, which represents the combined activation during overt speech production over the course of the scan. This contrast theoretically utilizes all aspects of language processing, including connected speech production (fluency),^30^ speech sound processing (phonology),^49^ and the controlled retrieval of information related to an object (semantics),^50^ the latter of which should rely on regions that are commonly used during semantic processing in both production and comprehension tasks.^51,52^ This single contrast was therefore likely to identify neural correlates of multiple language dimensions, while maximizing the amount of imaging data included and minimizing the multiple comparisons that would have occurred if multiple separate contrasts were used.

ImCalc was used to create a ‘mean activation image’ from the mean of each subject’s first and second time point ‘Speech + Count > Rest’ contrast images. These ‘mean activation’ images were entered into a second-level analysis assessing the main effect of group (patients versus controls) on regional activation during speech production (outlined below). ImCalc was also used to subtract each subject’s first timepoint ‘Speech + Count > Rest’ contrast image from the corresponding second time point contrast image to obtain an ‘activation change’ image representing the change in parameter estimate over time. These ‘activation change’ images were entered into second-level analyses with language change, i.e. the change over time of each of the three PC scores, as covariates (outlined below).

### Functional MRI second-level analysis

First-level ‘Speech + Count > Rest’ contrast estimates from each subject at each voxel were entered into group-level random-effects analyses in SPM12. Statistical thresholding used a voxel-wise cluster forming threshold of *P* < 0.005 (uncorrected) and a cluster-level threshold of *P* < 0.05 after familywise error correction. To minimize the confounding effect of variable lesion morphology, analyses were restricted to grey matter voxels in which no patient had a lesion. This resulted in a large proportion of the left hemisphere being excluded.

To identify whether regional activation during speech production varied between participant groups (patients versus controls) and time points (T1 versus T2), we performed a second-level mixed-design ANOVA using SPM’s ‘flexible factorial’ option with first-level ‘Speech + Count > Rest’ contrast estimate as the dependent variable, ‘time point’ as the within-subjects factor and ‘participant group’ as the between-subjects factor. This assessed for a Participant group × Time point interaction and a main effect of time point (T1 versus T2); because the error term for this model was within-subjects, a main effect of group was assessed using an independent sample *t*-test comparing the ‘mean activation images’ of patients versus controls.

To identify regions in which activation associated with language performance, patient group ‘Speech + Count > Rest’ contrast estimates were entered into second-level analyses with the three neuropsychological PCs included as regressors of interest. T1 brain activation was related to T1 PC1, T1 PC2 and T1 PC3 scores in three separate analyses; ‘activation change’ (obtained by subtracting each participant’s contrast image at T1 from T2) was associated with change over time in scores of each of the three PCs (PC1 change, PC2 change and PC3 change, obtained by subtracting each participant’s PC score at T1 from T2) in three separate analyses; and T2 activation was associated with T2 PC1, T2 PC2 and T2 PC3 scores in three separate analyses. In addition, we wanted to identify regions in which activation change was associated with language performance change after controlling for baseline severity of the language deficit. We therefore repeated second-level analyses in which activation change was associated with PC1 change, PC2 change and PC3 change, including T1 PC1, T1 PC2 and T1 PC3 scores as regressors of no interest in their respective separate design matrices.

We extracted the mean parameter estimate from each cluster identified as being associated with language performance in the above analyses using MarsBaR 0.44.^53^ Mean parameter estimate here represents either mean activation during ‘Speech + Count > Rest’ at T1 or T2 or the change in mean activation during ‘Speech + Count > Rest’ between T1 and T2. To account for the relationship between a given cluster-derived parameter estimate and language scores above and beyond that explained by cofounding factors, we used robust regression implemented in MATLAB 2018a to construct several analyses per cluster parameter estimate with language PC score as the dependent variable. The first model included mean parameter estimate alone. For clusters assessing activation change between T1 and T2, three additional models were constructed. The first additional model included mean activation change as well as T1 PC score. If mean activation change was significant in the first additional model, a second additional model added T1 PC × Mean activation change to check for a significant interaction, and a third additional model included the first or second additional model (depending on whether the interaction was significant) as well as lesion volume, years of education and age. Regression coefficients are reported as unstandardized.

Anatomical labels were defined using the Harvard–Oxford atlas for cortical regions^54^ and the Automated Anatomical Labelling atlas for subcortical regions.^55^

See the online Supplementary material for additional details regarding participants, neuropsychological testing, statistical analyses, functional MRI design and processing.

### Data availability

The data of this study are available upon reasonable request.

## Results

### Neuropsychological tests

At the group level, patients with aphasia were significantly impaired relative to controls on virtually all tests at T1 (Supplementary Table 4) but performed significantly better by T2 (Supplementary Table 5) such that their performance approached control levels (Supplementary Table 6).

### Principal component analysis of the neuropsychological scores

We performed varimax-rotated PCA on the correlation matrix of neuropsychological test scores of patients at T1. Three rotated PCs with eigenvalues greater than 1 were obtained (Table 1).^56^ PC1 was interpreted as representing fluency of connected speech; PC2 was interpreted as representing semantic/executive performance; and PC3 was interpreted as representing phonological ability (Table 1). These components strongly resemble the PCA structure that was previously obtained using independent data from acute^31^ and chronic post-stroke aphasia^26–28^ and which has been formally shown to be highly stable in chronic aphasia.^57^

**Table 1:** Component matrix of neuropsychological scores from patients with post-stroke aphasia at time point 1

Neuropsychological test	Component loadings		
	PC 1	PC 2	PC3
Cinderella ICWs per second	**0.93**	0.08	0.24
Cinderella syllables per second	**0.89**	0.12	0.23
Cinderella total ICWs	**0.86**	0.18	0.13
Cinderella NAS	**0.80**	0.27	0.44
CAT spoken picture description	**0.73**	0.21	0.46
CAT fluency	**0.64**	0.43	0.48
CAT spoken comprehension	0.17	**0.91**	0.25
CAT written comprehension	0.24	**0.83**	0.33
Decision task IES	0.10	**−0.76**	−0.10
Ravens	0.42	**0.69**	0.14
CAT cognitive	0.36	**0.61**	0.29
Speech task appropriate minus inappropriate ICWs	0.42	0.53	0.51
CAT repetition	0.22	0.16	**0.89**
CAT object naming	0.29	0.44	**0.78**
CAT reading	0.27	0.51	**0.72**
Speech task syllable rate	0.35	0.15	**0.67**

Varimax rotated PCA was performed on the neuropsychological scores of patients with post-stroke aphasia at time point 1 (2 weeks post-stroke). The loading of each score onto each rotated principal component is shown. Variables with major loadings (defined as >0.60) are in bold. CAT = Comprehensive Aphasia Test; IES = inverse efficiency score; NAS = Narrative Aphasia Score.

To enable direct comparisons between T1 and T2, the patients’ neuropsychological scores at T2 were back-projected into the ‘T1 PCA space’ (see Supplementary material). PC1 scores were significantly better at T2 than T1 [median −0.02 (interquartile range, IQR 1.20) at T1 versus 0.62 (0.96) at T2; Wilcoxon signed-rank test, *z* = 3.67, *P* < 0.0005]. Similarly, PC2 scores were significantly better at T2 than T1 [median 0.43 (IQR 1.06) at T1 versus 0.51 (0.64) at T2; Wilcoxon signed-rank test, *z* = 3.01, *P* = 0.003]. However, PC3 scores were not significantly better at T2 than T1 [median 0.21 (IQR 1.07) at T1 versus 0.26 (0.59) at T2; Wilcoxon signed-rank test, *z* = 0.88, *P* = 0.38]. Like the raw neuropsychological scores, the three PCs had a smaller IQR at T2 compared to T1. We therefore expected T1 PC scores to be negatively correlated with the change in performance over time, even if there were no underlying correlation between scores at T1 and T2.^32^ Indeed, T1 PC1 was significantly negatively correlated with PC1 change (Spearman’s rho = −0.55, *P* = 0.003); T1 PC2 was significantly negatively correlated with PC2 change (Spearman’s rho = −0.57, *P* = 0.002); and T1 PC3 was significantly negatively correlated with PC3 change (Spearman’s rho = −0.81, *P* = 5 × 10^−7^). It was therefore important to control for baseline PC score when considering the neural correlates of performance change. Otherwise, the regions might reflect change or simply the baseline performance itself.

If aphasia recovery occurred in a unidimensional manner, one would expect the different ‘PC change’ scores to be strongly, positively correlated. However, there was no significant correlation between PC1 change and PC2 change (Spearman’s rho = −0.18, *P* = 0.39) or between PC2 change and PC3 change (Spearman’s rho = 0.20, *P* = 0.32), while PC1 change was significantly negatively correlated with PC3 change (Spearman’s rho = −0.62, *P* = 0.001). These results suggest that aphasia recovery is multidimensional and raises the intriguing possibility that different components of language might recover in an anticorrelated manner, as better fluency recovery was associated with poorer phonological recovery in this sample.

**Figure 2:**
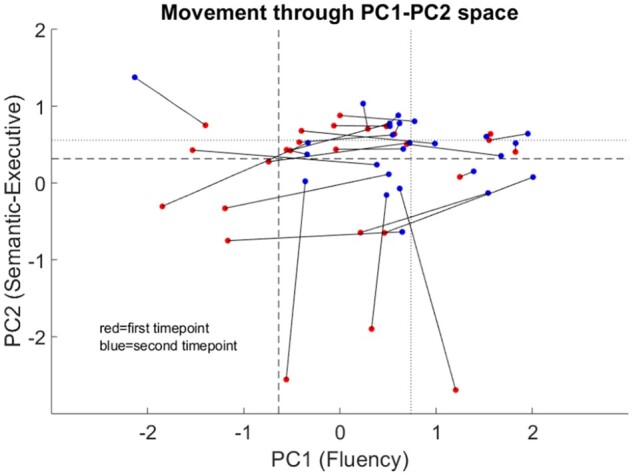
**Movement through PCA space during recovery.** Scatter plot depicting the 26 patients with post-stroke aphasia moving through PC1–PC2 space between 2 weeks and 4 months post-stroke. Each line represents an individual patient with the red circle their time point 1 performance (2 weeks post-stroke) and the blue circle their time point 2 performance (4 months post-stroke). Dashed lines represent the ‘lower bound of normal control performance’; dotted lines represent the ‘mean PC score’; *x-* and *y*-axes show the component scores. PC1 = ‘fluency’ principal component; PC2 = ‘semantic/executive’ principal component.

Figure 2 scatter plot depicts the 26 patients moving through PC1–PC2 space between 2 weeks and 4 months post-stroke (Fig. 2; see Supplementary Fig. 2 for PC1–PC3 and PC2–PC3 scatter plots). If there was a singular recovery process then the patients would move uniformly towards the upper, right-hand corner (towards control level performance). Instead, it is clear that the patients did not ‘move’ together through each PCA space, but rather had different recovery trajectories (Fig. 2).

### Regional activation during speech production

‘Speech + Count > Rest’ identified significant bilateral activation throughout frontal–temporo-parietal, insular and anterior cingulate cortex (Supplementary Fig. 3A and Supplementary Table 8). ‘Rest > Speech + Count’ identified significant bilateral deactivation during speech in core regions of the Default Mode Network (Supplementary Fig. 3B and Supplementary Table 8).^58^ Using a mixed-design ANOVA with first-level ‘Speech + Count > Rest’ contrast estimate as the dependent variable, ‘time point’ as the within-subjects factor and ‘participant group’ as the between-subjects factor, the main effect of time point (T1 versus T2) was not significant and the Participant group × Time point interaction was not significant. The main effect of group did not identify any regions of significantly greater activation in patients than controls. We found significantly less activation in patients than controls in three clusters, including one in the right posterior cingulate and temporo-parietal cortex (Supplementary Fig. 3C and Supplementary Table 9).

### Activation positively associated with fluency at 2 weeks post-stroke

**Figure 3:**
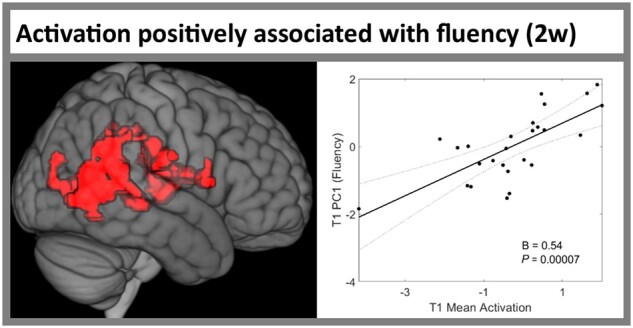
**Region in which activation was positively associated with fluency at 2 weeks post-stroke.** *Left*: The cluster from Supplementary Table 10 in which activation during ‘Speech + Count > Rest’ was positively associated with PC1 ‘fluency’ score in patients with post-stroke aphasia at 2 weeks post-stroke. *Right*: Scatter plot shows the significant, positive association between mean activation in this cluster (*x*-axis) and PC1 ‘fluency’ score at 2 weeks post-stroke (*y*-axis) from the robust regression model in Supplementary Table 11. Statistical thresholding used a voxel-wise cluster-forming threshold of *P* < 0.005 (uncorrected) and a cluster-level threshold of *P* < 0.05 after familywise error correction. B = unstandardized regression coefficient; PC1 = ‘fluency’ principal component; T1 = time point 1 (2 weeks post-stroke).

We did not identify any clusters in which T1 activation was associated with T1 ‘semantic/executive’ (PC2) or ‘phonology’ (PC3) score. However, T1 activation in a cluster in the right posterior supramarginal gyrus, insular cortex and temporo-occipital middle temporal gyrus (MTG) was positively associated with T1 ‘fluency’ (PC1) score (Fig. 3 and Supplementary Table 10). Mean activation extracted from this cluster was significantly positively associated with T1 PC1 score (beta = 0.54, *P* = 7.1 × 10^−5^), even after including lesion volume, years of education and age (beta = 0.51, *P* = 0.0004) (Fig. 3 and Supplementary Table 11).

### Activation change positively associated with fluency improvement between 2 weeks and 4 months post-stroke

**Figure 4:**
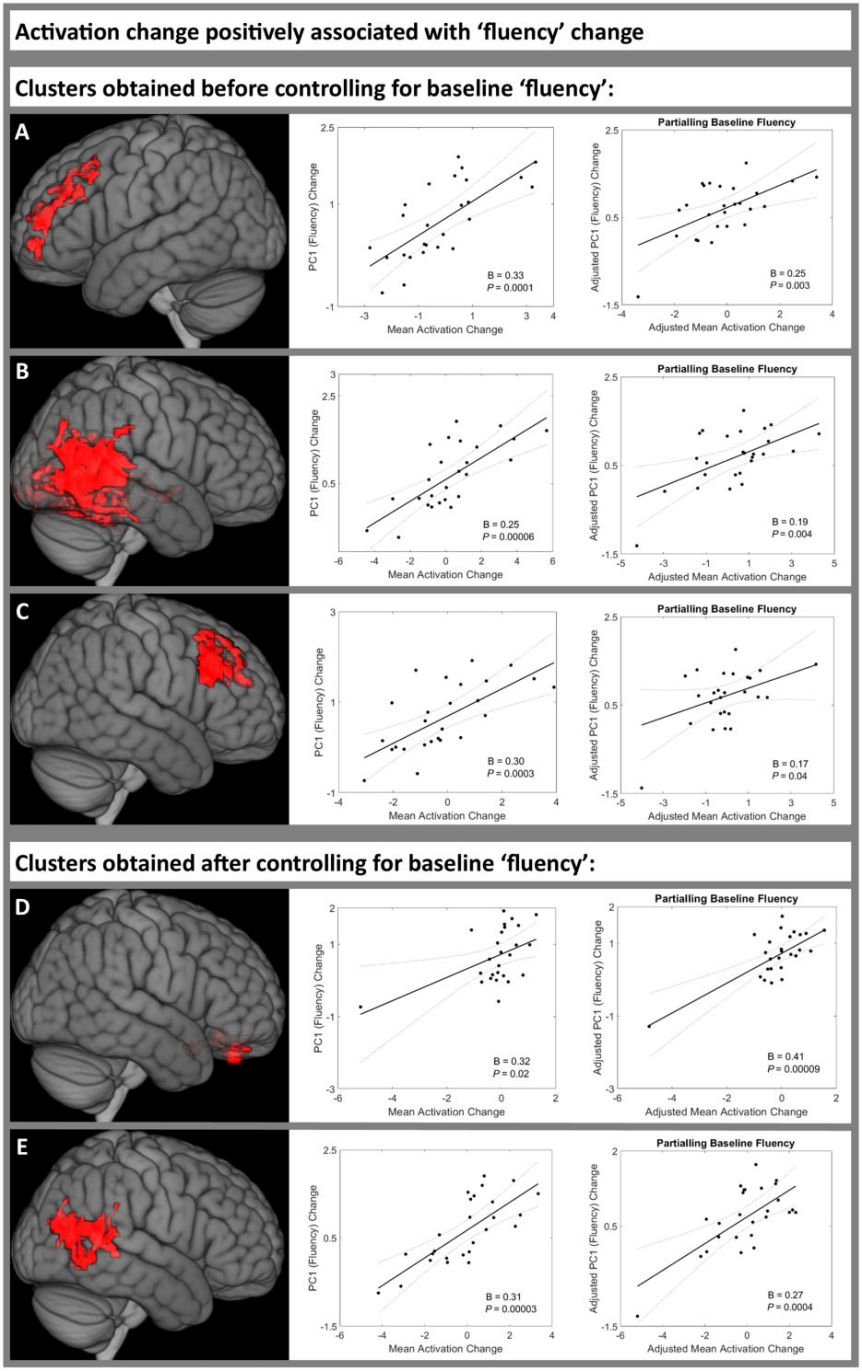
**Regions in which increased activation was positively associated with fluency improvement between 2 weeks and 4 months post-stroke.** Activation change was positively associated with PC1 ‘fluency’ score change between time point 1 (2 weeks) and time point 2 (4 months post-stroke) in patients with post-stroke aphasia. Three clusters were significant before controlling for baseline ‘fluency’ score (**A**–**C**) (Supplementary Tables 12 and 13), two clusters were significant after controlling for baseline ‘fluency’ score (**D** and **E**) Supplementary Tables 14 and 15. *Left*: Each cluster rendered on the MNI standard brain template. *Middle*: The association between the mean activation change of each cluster and PC1 ‘fluency’ score change, using robust regression. *Right*: The association between the mean activation change of each cluster and PC1 ‘fluency’ score change, controlling for baseline ‘fluency’ score, using robust regression. Statistical thresholding used a voxel-wise cluster-forming threshold of *P* < 0.005 (uncorrected) and a cluster-level threshold of *P* < 0.05 after familywise error correction. B = unstandardized regression coefficient; PC1 = ‘fluency’ principal component; T1 = time point 1 (2 weeks post-stroke); T2 = time point 2 (4 months post-stroke).

Before including baseline ‘fluency’ score (PC1) in the mass univariate analysis, activation change in three clusters was significantly positively associated with PC1 improvement (Supplementary Table 12). The first cluster was centred in the left middle frontal gyrus (MFG; Fig. 4A); the second cluster was centred in the right temporo-occipital MTG (Fig. 4B), in a location similar to the cluster associated with PC1 score at T1 (Fig. 3); and the third cluster was in the right MFG (Fig. 4C). Mean activation change extracted from each of these clusters was significantly positively associated with PC1 improvement both before and after including T1 PC1 score, and even after including lesion volume, years of education and age (cluster 1 beta = 0.29, *P* = 0.0003; cluster 2 beta = 0.16, *P* = 0.04; cluster 3 beta = 0.30, *P* = 0.0001) (Fig. 4 and Supplementary Table 13).

After including baseline ‘fluency’ (PC1) score in the mass univariate analysis, activation change in two clusters was significantly positively associated with better PC1 improvement (Supplementary Table 14). The first cluster was centred in the bilateral ventromedial prefrontal cortex (Fig. 4D). The second cluster was centred in the right temporooccipital MTG (Fig. 4E), in a location similar to the clusters associated with PC1 at T1 (Fig. 3) and with PC1 change before including T1 PC1 in the mass univariate analysis (Fig. 4B). Mean activation change extracted from both clusters was significantly positively associated with PC1 improvement both before and after including T1 PC1 score, and even after including lesion volume, years of education and age (cluster 1 beta = 0.38, *P* = 0.0001; cluster 2 beta = 0.23, *P* = 0.003; Fig. 4 and Supplementary Table 15).

We did not identify any clusters in which activation change was significantly negatively associated with ‘fluency’ PC1 change.

### Activation change negatively associated with semantic/executive change between 2 weeks and 4 months post-stroke

We did not identify any clusters in which activation change was significantly positively associated with PC2 change.

**Figure 5:**
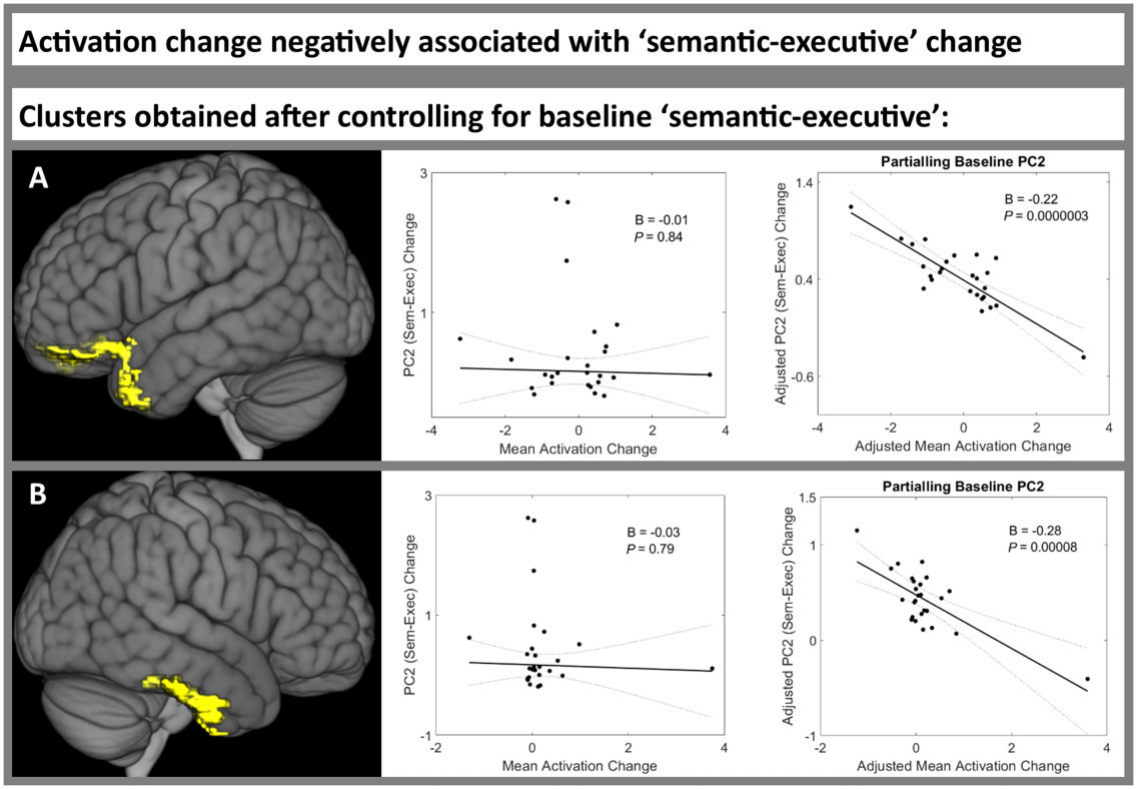
**Regions in which increased activation was negatively associated with semantic/executive improvement between 2 weeks and 4 months post-stroke.** Activation change was negatively associated with PC2 ‘semantic/executive’ score change between time point 1 (2 weeks) and time point 2 (4 months post-stroke) in patients with post-stroke aphasia. No clusters were significant before controlling for baseline ‘semantic/executive’ score, but two clusters were significant after controlling for baseline ‘semantic/executive’ score (**A** and **B**) (Supplementary Tables 16 and 17). *Left*: Each cluster rendered on the MNI standard brain template. *Middle*: The association between the mean activation change of each cluster and PC2 ‘semantic/executive’ score change, using robust regression. *Right*: The association between the mean activation change of each cluster and PC2 ‘semantic/executive’ score change, controlling for baseline ‘semantic/executive’ score, using robust regression. Statistical thresholding used a voxel-wise cluster forming threshold of *P* < 0.005 (uncorrected) and a cluster-level threshold of *P* < 0.05 after familywise error correction. B = unstandardized regression coefficient; PC2 = ‘semantic/executive’ principal component; T1 = time point 1 (2 weeks post-stroke); T2 = time point 2 (4 months post stroke).

Before including baseline ‘semantic/executive’ (PC2) score in the mass univariate analysis, no clusters were significantly negatively associated with PC2 change. However, after including baseline PC2 score in the mass univariate analysis, activation change in two clusters was significantly negatively associated with PC2 change (Supplementary Table 16). The first cluster was in the left temporal pole, frontal medial cortex and frontal pole (Fig. 5A). The second cluster encompassed the right temporal pole, anterior MTG and posterior inferior temporal gyrus (Fig. 5B). These two clusters therefore encompass core regions of the bilateral semantic network.^50^ For both clusters, extracted mean activation change was not associated with PC2 change before including T1 PC2 score (cluster 1 beta = −0.01, *P* = 0.84; cluster 2 beta = −0.03, *P* = 0.79; Fig. 5 and Supplementary Table 17). However, after including T1 PC2 score, ‘mean activation change’ became significantly negatively associated with PC2 change in both clusters (cluster 1 beta = −0.22, *P* = 2.5 × 10^−7^; cluster 2 beta = −0.28, *P* = 8.0 × 10^−5^; Fig. 5 and Supplementary Table 17). This remained true after adding lesion volume, years of education and age (cluster 1’s beta = −0.22, *P* = 3.0 × 10^−6^; cluster 2’s beta = −0.29, *P* = 5.2 × 10^−5^; Supplementary Table 17). These results demonstrate that including baseline language performance in the mass univariate analysis can identify novel areas that are associated with language change but would otherwise remain obscured.

### Activation change negatively associated with phonology change between 2 weeks and 4 months post-stroke

We did not identify any clusters in which activation change was significantly positively associated with ‘phonology’ (PC3) change.

**Figure 6:**
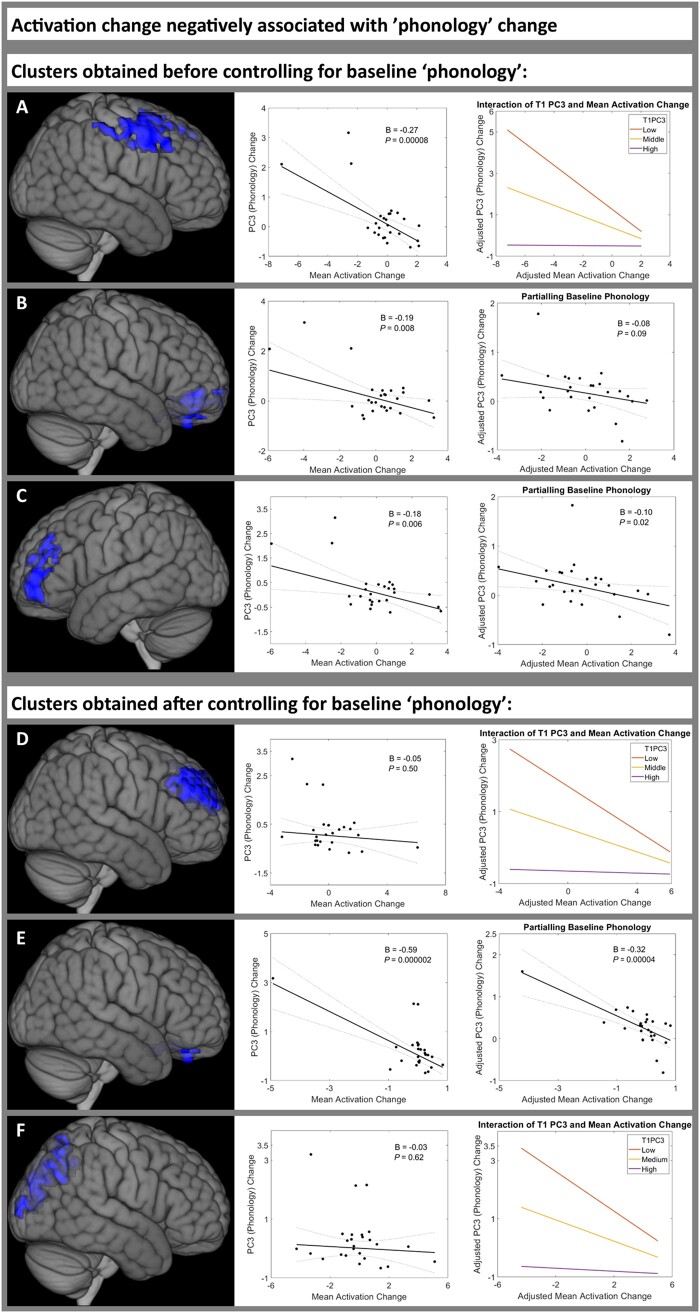
**Regions in which increased activation was negatively associated with phonology improvement between 2 weeks and 4 months post-stroke.** Activation change was negatively associated with PC3 ‘phonology’ score change between time point 1 (2 weeks) and time point 2 (4 months post-stroke) in patients with post-stroke aphasia. Three clusters were significant before controlling for baseline ‘phonology’ score (**A**–**C**) (Supplementary Tables 18 and 19), three clusters were significant after controlling for baseline ‘phonology’ score (**D**–**F**) (Supplementary Tables 20 and 21). *Left*: Each cluster rendered on the MNI standard brain template. *Middle*: The association between the mean activation change of each cluster and PC3 ‘phonology’ score change, using robust regression. For clusters **B**, **C** and **E**, the *right* column shows the association between the mean activation change of each cluster and PC3 ‘phonology’ score change, controlling for baseline ‘phonology’ score, using robust regression. For clusters **A**, **D** and **F**, the *right* column shows the interaction plot for the adjusted association between the mean activation change of each cluster and PC3 ‘phonology’ score change, with baseline ‘phonology’ score fixed at high, middle and low values, using robust regression. Statistical thresholding used a voxel-wise cluster forming threshold of *P* < 0.005 (uncorrected) and a cluster-level threshold of *P* < 0.05 after familywise error correction. B = unstandardized regression coefficient; PC3 = ‘phonology’ principal component; T1 = time point 1 (2 weeks post-stroke); T2 = time point 2 (4 months post-stroke).

Before including baseline PC3 in the mass univariate analysis, activation change in three clusters was significantly negatively associated with PC3 change (Supplementary Table 18). The first cluster affected the bilateral precentral gyrus, MFG and superior frontal gyrus (Fig. 6A). Mean activation change extracted from the first cluster was significantly negatively associated with PC3 change (beta = −0.27, *P* = 8.3 × 10^−5^); however, when including T1 PC3 score and T1 PC3 × Mean activation change in the model, it transpired that there was a significant main effect of T1 PC3 (beta = −0.49, *P* = 6.9 × 10^−5^) and a significant interaction (T1 PC3 × Mean activation change beta = 0.14, *P* = 0.0009) such that ‘mean activation change’ was only negatively associated with PC3 change in patients with low PC3 scores at T1 (Fig. 6A and Supplementary Table 19). The second cluster was in bilateral ventromedial prefrontal cortex (Fig. 6B), similar to the clusters that were positively associated with PC1 change (Fig. 4D) and negatively associated with PC2 change (Fig. 5A). However, mean activation change extracted from cluster 2 was not significantly associated with PC3 change after including T1 PC3 (beta = −0.08, *P* = 0.09; Fig. 6B and Supplementary Table 19). The third cluster was in the left frontal pole (Fig. 6C), partially overlapping with the cluster positively associated with PC1 change (Fig. 4A). However, mean activation change extracted from cluster 3 was not significantly negatively associated with PC3 change after including T1PC3, lesion volume, years of education and age (beta = −0.11, *P* = 0.08; Fig. 6C and Supplementary Table 19).

After including baseline ‘phonology’ (PC3) score in the mass univariate analysis, activation change in three clusters was significantly negatively associated with PC3 change (Supplementary Table 20). The first cluster affected the bilateral frontal poles and midline superior frontal gyrus (Fig. 6D), more medially than the bilateral MFG clusters positively associated with PC1 change (Fig. 4A and C). Mean activation change extracted from cluster 1 was not associated with PC3 change before including T1 PC3 (beta = −0.05, *P* = 0.50), but was negatively associated with PC3 change after including T1 PC3 (beta = −0.13, *P* = 0.0006) and had a significant T1 PC3 × Mean activation change interaction (T1 PC3 × Mean activation change beta = 0.08, *P* = 0.05) such that ‘mean activation change’ was only negatively associated with PC3 change in patients with low PC3 scores at T1 (Fig. 6D and Supplementary Table 21). The second cluster affected the ventromedial prefrontal cortex (Fig. 6E), similar to the clusters that were positively associated with PC1 change (Fig. 4D) and negatively associated with PC2 change (Fig. 5A). Mean activation change extracted from cluster 2 was negatively associated with PC3 change both before and after including T1 PC3 score, and even after including lesion volume, years of education and age (beta = −0.28, *P* = 0.0001; Fig. 6E and Supplementary Table 21). The third cluster was in the precuneus (Fig. 6F). Mean activation change extracted from cluster 3 was negatively associated with PC3 change before and after including T1 PC3 (model including T1 PC3 and interaction term, beta = −0.15, *P* = 0.0002) and had a significant T1 PC3 × Mean activation change interaction (T1 PC3 × mMean activation change beta = 0.09, *P* = 0.03) such that ‘mean activation change’ was only negatively associated with PC3 change in patients with low PC3 scores at T1 (Fig. 6F and Supplementary Table 21).

We did not identify any clusters in which T2 activation was associated with T2 PC1, PC2 or PC3 score.

## Discussion

This study investigated the nature of aphasia recovery post-stroke by analysing data from one of the largest longitudinal, combined neuropsychological and functional neuroimaging studies in the subacute stage of aphasia recovery to date. The results show for the first time that (i) behavioural variation is multidimensional at the subacute phase; (ii) recovery is also multidimensional in nature and does not reflect a single, unitary factor; and (iii) these multidimensional shifts in performance are related to changes in task-induced activation in different brain regions. Moreover, from a methodological perspective, the study also showed that investigations of recovery need to account for baseline performance to avoid potential false positive and negative findings. Overall, this work provides novel insights into the neural basis of aphasia recovery, and suggests that neuromodulatory treatments targeting the same neural region in all aphasic stroke survivors might be ineffective or even impair recovery, and should instead be tailored depending on the specific language profile of each individual patient.

First, considering the cross-sectional neuropsychological data from the subacute phase, we found that the post-stroke aphasia language variations can be dissociated into orthogonal principal components. Aligning with previous research,^31^ the observed PCA structure consisted of fluency (PC1), semantic/executive function (PC2) and phonology (PC3). These behavioural factors also directly mirror the core PCA structure obtained in previous investigations of post-stroke aphasia patients in the chronic phase.^26–28,57,59^ This suggests that, even though patients’ performance improves over time, the behavioural variation in both the subacute and chronic phases can be captured by the same graded dimensions (phonology, fluency, semantics and executive skill).

A central aim of this study was to investigate whether these distinct underlying components of language have different recovery trajectories. The clear and novel answer was that recovery is not a single monolithic factor, but rather that the distinct language components recovered in either uncorrelated (for fluency versus semantic/executive and semantic/executive versus phonology) or anticorrelated (for fluency versus phonology) ways. This result suggests that aphasia recovery is heterogeneous and multidimensional. Consequently, aphasia treatment strategies and assessment of their efficacy should avoid single targets and outcome measures, and instead consider multiple, distinct aspects of language. This alternative approach might improve treatment outcomes and make efficacy measures more sensitive. It would also ensure that potential negative trade-offs between recovery of different language components are not overlooked.

A third key finding was that these different underlying language components are associated with changing activation in multiple, primarily non-overlapping regions during aphasia recovery. These associations can be positive or negative depending on the language component in question. After controlling for baseline performance, demographic and clinical variables: fluency recovery was associated with increasing activation in bilateral MFG and right temporooccipital MTG/supramarginal gyrus; semantic/executive recovery was associated with reducing activation in bilateral ATLs; while phonology recovery was associated with reducing activation in precentral gyri, dorso-medial frontal poles and the precuneus. Overlapping clusters in the ventromedial prefrontal cortex were positively associated with fluency recovery but negatively associated with semantic/executive and phonology recovery. Thus, different aspects of language might rely on different neural regions for recovery. Unfortunately, clinical trials of non-invasive brain stimulation frequently target the same neural region in all stroke survivors regardless of their aphasia profile.^60–63^ This study suggests that doing so might be entirely ineffective or even impair recovery, depending on the specific language profile of each individual patient. Similarly, meta-analyses of non-invasive brain stimulation in post-stroke aphasia tend to ignore the aphasia profile or stimulation site of included studies.^64,65^ Unless these are considered, potential benefits or harms of non-invasive brain stimulation might be overlooked.

This study was also able to explore an important methodological issue: one should control for baseline language performance when identifying neuroimaging correlates of aphasia recovery. This follows from the fact that, when task performance/PCA factor scores have greater variance in the acute than more chronic phases (e.g. when performance approaches ceiling, *cf*. control performance levels), then the change in the score is inescapably anti-correlated with the score in the acute phase.^32^ With this in mind, we found little evidence for ‘false positives’ in the present study; most identified functional MRI clusters were still significantly associated with PC change after controlling for T1 PC score. However, we did find multiple examples of ‘false negatives’, in which a true association between activation change and PC change was masked by varying baseline language performance. Strikingly, no regions were associated with semantic/executive change before controlling for T1 score, yet bilateral ATL clusters were negatively associated with semantic/executive recovery after controlling for T1 score.

An unexpected implication of this study is that activation changes during the subacute phase of aphasia recovery may not be as large as previously suggested, at least for patients with mild–moderate aphasia.^12,15–19,21,66^ Specifically, we found no significant group-level activation differences between T1 and T2, despite significant behavioural improvement in 14 of the 16 neuropsychological tests. It is possible that this absence of group-level activation changes might be another consequence of the multidimensional nature of aphasia recovery. If recovery in different aspects of language relies on changing activation in different neural regions, then the resultant heterogeneity in activation changes will be much less likely to generate a single, homogeneous change when the data are combined simply at the group level.

Previous studies have reported regions of hyperactivation in post-stroke aphasia relative to controls at subacute time points post ictus.^18,19,21^ We only found that, averaged across T1 and T2, patients hypoactivated the left posterior cingulate/precuneus, right thalamus and right temporo-parietal cortex. Less activation in the right inferior parietal part of this hypoactivated cluster was associated with poorer fluency at T1, while normalisation of activation was associated with better fluency recovery. This suggests that ‘functional diaschisis’^67^ in right inferior parietal cortex might contribute to fluency deficits, and their resolution, post-stroke. Although inferior parietal and posterior temporal regions are not classically associated with fluency, one model postulates that an efference copy of speech is sent from premotor to inferior parietal and posterior temporal regions for comparison with perceived auditory information and optimization of ongoing speech.^68^ Indeed, the right posterior superior temporal sulcus plays a role during speech production in controls^69^; changing grey matter integrity in the right posterior MTG has been associated with changing object naming in chronic aphasia^70^; and grey matter volume in the right temporoparietal cortex has been associated with spontaneous speech, naming and repetition scores in chronic aphasia.^71^

Our results have wider implications for the mechanisms that might underlie aphasia recovery.

First, increasing and decreasing activation in specific regions of both hemispheres can be associated with recovery of different aspects of language. This is inconsistent with simplified left versus right ‘regional hierarchy’ models wherein engagement of the right hemisphere is suboptimal to left-dominant activation or might even be maladaptive through ‘transcallosal disinhibition’.^72,73^ Instead, it is consistent with a model in which healthy language relies on a bilateral albeit asymmetric left-biased network that can support at least partial recovery through upregulation of function in perilesional and contralateral areas (for a recent computational implementation see Chang and Lambon Ralph^74^).

Second, activation change in the ventromedial prefrontal cortex, dorso-medial frontal poles and precuneus was negatively correlated with phonological recovery. These clusters overlapped with regions deactivated in controls; thus, greater deactivation in task-negative regions was associated with better phonological recovery. This is consistent with previous work demonstrating that greater differential activity between the default mode network and task-positive networks was associated with better language performance post-stroke.^8^

Third, activation change in bilateral MFG was positively associated with fluency recovery, yet these regions were not activated in controls performing the same task. One would need controls to perform a more difficult language task to know whether these regions represent spare capacity that is upregulated through variable neuro-displacement both premorbidly (when task difficulty increases) and after stroke.^5,20^ A network including the bilateral MFG was activated during the ‘Decision’ task in controls,^33^ suggesting their involvement in domain-general executive processing.^20^ It is unclear why increased activity in domain-general regions was associated with fluency recovery but not semantic/executive recovery. A possible explanation is that the ‘Speech + Count > Rest’ contrast used in this analysis favours activations related to fluency, and the degree of recruitment of domain-general regions to a fluency task might not necessarily relate to the degree of recruitment of domain-general regions to tasks favouring semantic/executive function. Future work using separate fluency, semantic/executive and phonological functional MRI tasks would be needed to investigate this further.

Finally, activation change in the bilateral ATLs was negatively associated with semantic/executive recovery. These regions have been proposed to comprise the ‘hub’ of a distributed network subserving semantic representation.^50^ Previous research has suggested that variable neuro-displacement enables intrinsic spare capacity within the bilateral ATLs to ameliorate semantic impairment following damage/stimulation to one part of the system.^75–77^ The negative association between activation change and semantic/executive recovery might reflect the fact that individuals with deteriorating efficiency of the distributed semantic network (and thus worse semantic/executive recovery) need to activate their ‘hub’ more to perform a given semantic task,^78,79^ just as ATL activations in healthy participants tend to be higher for more demanding concepts or semantic tasks.^77^

This study has several limitations. First, the specific neural regions that were associated with recovery on different language components in this study should be replicated in an independent sample and assessed regarding how well they predict recovery in individuals with aphasia. Second, analyses were restricted to voxels in which no patient had a lesion, in order to remove any direct confounding effect of variable lesion morphology. Consequently, we might have missed positive associations between language performance and left hemisphere activation, as has been shown previously.^12^ However, most previous studies reporting such a compensatory role for left hemisphere language activation have tended to overlook variable lesion morphology as a confounding factor,^21,80–82^ or analysed restricted subgroups of patients with lesions confined to certain locations^12,16^ which does not completely account for voxel-wise variability in lesion location throughout the entire left hemisphere. Third, previous work using independent component analysis identified that propositional and non-propositional speech can have opposite effects on activation in the same spatiotemporal networks in the left versus right hemispheres.^33^ Although we observed bilateral fronto-temporo-parietal activation and identified neural correlates for all three language components using ‘Speech + Count > Rest’, our use of this contrast of interest and the mass univariate analysis method does mean that we are unable to say which aspects of propositional or non-propositional speech, and which distinct spatiotemporal networks, might be contributing to any of the identified neural correlates of language change or to the observed negative associations between activation and PC2/PC3.

## Conclusions and future directions

Our findings demonstrate that distinct underlying components of language have different recovery trajectories associated with changing activation in distinct neural regions. Targeting the same neural region in all aphasic stroke survivors might be ineffective or even impair recovery, depending on the specific language profile of each individual patient. As noted above, given the significant clinical challenges that arise immediately after stroke, most studies including the current one tend to recruit patients with mild–moderate aphasia. Future studies are needed to explore the recovery profiles and their neural correlates in more severely affected patients.

## Supplementary Material

awab377_Supplementary_Data

## Abbreviations

ATL: anterior temporal lobe
ICW: information carrying word
MFG: middle frontal gyrus
MTG: middle temporal gyrus
PC: principal component
PCA: principle component analysis
T1: time point 1
T2: time point 2
