# Supplementary material for: The multidimensional nature of aphasia recovery post-stroke
Source: Brain. 2022 Mar 10;145(4):1354–67. doi: 10.1093/brain/awab377 (PMC9128817; doi:10.1093/brain/awab377)
Supplement: awab377_Supplementary_Data [file awab377_supplementary_data.zip › brain-2021-00607-File009.pdf]

Supplementary Methods

Participants

One of the patients was left handed but had clear language deficits following stroke suggesting pre-morbid left hemisphere dominance for language; three had additional small infarcts (0.5, 1.5 and 3.5cm^3) in the right hemisphere but of the two whose right sided infarcts predated their left sided stroke, there was no history of ongoing clinical deficit. We included patients with any aphasia severity post-stroke, including those with mild deficits, to enable us to capture the full multidimensional profile of post-stroke aphasia. Three patients had NIH Stroke Scale scores of 0 at 2 weeks post-stroke (Supplementary Table S1) despite having ongoing language difficulties, in keeping with the low sensitivity of the NIH Stroke Scale at detecting mild aphasia.[1]

Neuropsychological tests

A subset of the neuropsychological measures tested in patients were available in the controls at both timepoints (‘Cinderella ICWs Per Second’, ‘Cinderella Syllables Per Second’, ‘Cinderella Total ICWs’, ‘Cinderella NAS’, ‘Decision Task IES’, ‘Speech Task Appropriate ICWs minus Inappropriate ICWs’, ‘Speech Task Syllable Rate’, ‘Ravens’, ‘CAT Cognitive’, and ‘CAT Fluency’).

Each Cinderella story assessment of connected speech production was transcribed from audio recordings and analysed to provide a Narrative Aphasia Score (NAS). This was an adaptation of the Comprehensive Aphasia Test (CAT) picture description scoring method,[2] which did not penalise pure articulatory or dysarthric errors. The NAS was calculated using the following formula:

NAS = ((Appropriate – Inappropriate Information Carrying Words)/Number of utterances) + Syntactic variety + Grammatical well-formedness

Statistical tests

All variables were assessed for normality using the Kolmogorov-Smirnov test. Group differences between patients at T1 and controls were performed using independent samples *t*-tests and Mann-Whitney U tests for normally and non-normally distributed variables, respectively. Changes over time between T1 and T2 were assessed using paired *t*-tests and Wilcoxon signed-rank tests for normally and non-normally distributed variables, respectively.

Principal Components

Scores from Principal Components (PCs) with an eigenvalue greater than one were taken to be estimates of underlying cognitive components. To enable direct comparisons to be made between T1 and T2, the patients' scores need to be in the same PCA 'space'. As the T2 data was not appropriate for PCA reduction (see main manuscript), the neuropsychological scores at T2 were back-projected into the 'T1 PCA space' to estimate each patient's PC score had their T2 scores been obtained at T1. This was done by Z-scoring each T2 score using the mean and SD at T1 before multiplying by the component-specific coefficients obtained from the T1 PCA and summating across scores for that participant.

For each PC, a 'lower bound of normal PC score' was calculated, which was taken to be the set of PC scores that would be obtained if a hypothetical individual scored exactly at the fifth percentile of normal on each of the 16 neuropsychological tests. The fifth percentile of normal on each of the 16 neuropsychological tests was estimated using the neuropsychological data obtained from controls in this study, or for tests not performed in the controls of this study, that were published in the CAT manual.[2] The fifth percentile was estimated from control data as:

Fifth percentile = sample mean – (*t*-statistic * sample SD)

Where the *t*-statistic value was for the number of degrees of freedom (n-1) to give a cumulative probability of 0.05 of the control performance being worse than that value.

For each PC, a 'mean PC score' was also calculated, which would have been obtained if an individual scored at the control sample mean on each of the 16 neuropsychological tests at T1. The control sample mean on each of the 16 neuropsychological tests was estimated using the neuropsychological data obtained from controls in this study, or for tests not performed in the controls of this study, that were published in the CAT manual.[2]

Functional MRI data acquisition

A Siemens Magnetom Trio 3T scanner was used to obtain a 1mm^3 T_1-weighted image with field maps, and a whole-brain T_2*-weighted, gradient echoplanar fMRI sequence consisting of 36 axial slices acquired in an interleaved order (resolution 3.5x3.5x3.0mm^3; slice thickness 3mm; field of view 225x225x108mm; repetition time 10s; acquisition time 2s; second echo time 31ms; flip angle 90).

Functional MRI preprocessing

EPI volumes were initially preprocessed using FMRIB Software Library (www.fmrib.ox.ac.uk/fsl)[3] to perform: motion correction with MCFLIRT[4]; non-brain removal using BET[5]; spatial smoothing using an 8mm full-width at half maximum Gaussian kernel; and highpass temporal filtering with cutoff 100s. Statistical Parametric Mapping (SPM) 12 (Wellcome Centre for Human Neuroimaging, London UK; www.fil.ion.ucl.ac.uk/spm/) was used to: coregister the mean of run one to the T_1-weighted image in native space before applying this to all EPI volumes; normalise the T_1-weighted image into Montreal Neurological Institute (MNI) space using a modified unified segmentation-normalisation procedure optimised for focally lesioned brain[6]; and apply the same normalisation transformation to the coregistered EPI volumes. T_1-weighted images

for the same patient were normalised separately at T1 and T2, as structural deformation can occur over time in regions remote from the stroke,[7] which could alter the transformation required to map the same patient's image into standard space.

Functional MRI first-level analysis

The canonical HRF without spatial or temporal derivatives was used as it has been validated in sparsely sampled data[8] and has been used previously for analysis of sparsely sampled fMRI data during overt speech production.[9, 10] SPM's FAST pre-whitening option was used to account for temporal autocorrelation.[11]

Functional MRI second-level analysis

A binary lesion mask was manually delineated in each patient's native space at both timepoints by a neurologist (FG). Each lesion mask at T1 was transformed into MNI space using the normalisation transformation obtained previously. ImCalc was used to binarise the overlap of all patients' masks in MNI space and subtract this binarised lesion overlap from the SPM MNI grey matter template. The resultant mask, indicating all grey matter voxels in which no patient had a lesion in MNI space, was used for pre-threshold masking of those patient group second-level analyses which incorporated a language regressor of interest.

References

1. Grönberg A, Henriksson I, Lindgren A. Accuracy of NIH Stroke Scale for diagnosing aphasia. *Acta Neurol Scand*. 2021;143(4):375-382.
2. Swinburn K, Baker G, Howard D. *CAT: the comprehensive aphasia test*. Psychology Press; 2005.
3. Jenkinson M, Beckmann CF, Behrens TE, Woolrich MW, Smith SM. FSL. *Neuroimage*. 2012;62(2):782-90.
4. Jenkinson M, Bannister P, Brady M, Smith S. Improved optimization for the robust and accurate linear registration and motion correction of brain images. *Neuroimage*. 2002;17(2):825-41.
5. Smith SM. Fast robust automated brain extraction. *Human Brain Mapping*. 2002;17(3):143-155.
6. Seghier ML, Ramlackhansingh A, Crinion J, Leff AP, Price CJ. Lesion identification using unified segmentation-normalisation models and fuzzy clustering. *Neuroimage*. 2008;41(4):1253-1266.
7. Buffon F, Molko N, Herve D, et al. Longitudinal diffusion changes in cerebral hemispheres after MCA infarcts. *Journal of Cerebral Blood Flow and Metabolism*. 2005;25(5):641-650.
8. Perrachione TK, Ghosh SS. Optimized design and analysis of sparse-sampling fMRI experiments. *Frontiers in Neuroscience*. 2013;755.

9. Correia JM, Caballero-Gaudes C, Guediche S, Carreiras M. Phonatory and articulatory representations of speech production in cortical and subcortical fMRI responses. *Sci Rep*. 2020;10(1):4529.
10. Simmonds AJ, Leech R, Collins C, Redjep O, Wise RJ. Sensory-motor integration during speech production localizes to both left and right plana temporale. *J Neurosci*. 2014;34(39):12963-72.
11. Olszowy W, Aston J, Rua C, Williams GB. Accurate autocorrelation modeling substantially improves fMRI reliability. *Nat Commun*. 2019;10(1):1220.
