# Supplementary material for: The multidimensional nature of aphasia recovery post-stroke
Source: Brain. 2022 Mar 10;145(4):1354–67. doi: 10.1093/brain/awab377 (PMC9128817; doi:10.1093/brain/awab377)
Supplement: awab377_Supplementary_Data [file awab377_supplementary_data.zip › brain-2021-00607-File010.pdf]

Supplementary Results

Neuropsychological tests

Paired statistical tests confirmed that patients performed significantly better at 4 months (by T2) than 2 weeks (T1) post-stroke on all 16 measures, except for 'Decision Task IES' and 'Speech Task Syllable Rate' (Supplementary Table S5) where their performance remained the same. Consequently, patient performance at T2 started to approach control levels, with 'CAT Cognitive' and 'Decision Task IES' no longer being significantly impaired compared to the controls (Supplementary Table S6). Each of the 16 neuropsychological tests had a smaller interquartile range (IQR) at T2 than T1 (Supplementary Table S5).

Principal Component Analysis of the neuropsychological scores

Supplementary Figure S1: Scree plot from Principal Component Analysis of neuropsychological scores from patients with post-stroke aphasia at Timepoint 1

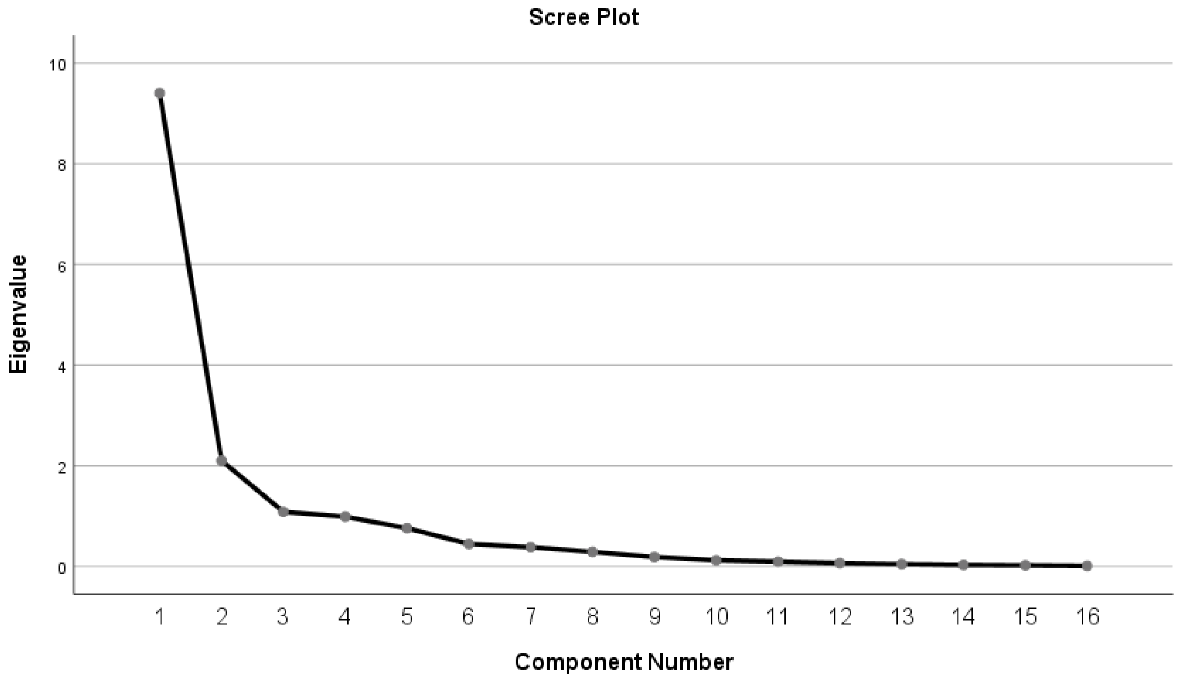

Supplementary Figure S1 Legend: Scree plot obtained from the Principal Component Analysis of the correlation matrix of neuropsychological test scores from patients with post-stroke aphasia at Timepoint 1 (2 weeks post-stroke).

The scree plot has an inflection point after three PCs (Supplementary Figure S1), confirming that 3 components should be retained.[1] The Kaiser-Meyer-Olkin value for the Timepoint 1 Principal Component Analysis was 0.79, indicating adequate sampling.[2] Bartlett's test of sphericity was significant (approximate χ^2_{120}=465, p=4x10^{-42}), suggesting these data could be factorised. Three rotated principal components (PCs) with eigenvalues greater than 1

explaining 30.45% (PC1), 25.37% (PC2) and 22.84% (PC3) of the variance were obtained (Table 1).

PC1 was primarily loaded onto by measures of connected speech production obtained from analysis of the Cinderella story (‘Cinderella ICWs Per Second’, ‘Cinderella Syllables Per Second’, ‘Cinderella Total ICWs’ and ‘Cinderella NAS’) as well as ‘CAT Spoken Picture Description’ and ‘CAT Fluency’. Thus, PC1 was interpreted as representing fluency of connected speech (Table 1). PC2 was loaded onto primarily by ‘CAT Spoken Comprehension’, ‘CAT Written Comprehension’, ‘CAT Cognitive’, ‘Ravens’ and in-scanner ‘Decision Task IES’ (lower scores representing better performance thus ‘Decision Task IES’ loaded negatively onto PC2). Thus, PC2 was interpreted as representing semantic/executive performance (Table 1). PC3 was loaded onto primarily by performance on ‘CAT Repetition’, ‘CAT Object Naming’, ‘CAT Reading’ and in-scanner ‘Speech Task Syllable Rate’. Thus, PC3 was interpreted as representing phonological ability (Table 1). This PCA structure has been replicated across different research groups.

We also performed varimax-rotated PCA on the correlation matrix of neuropsychological test scores of patients at T2. The Kaiser-Meyer-Olkin value was 0.46, indicating inadequate sampling.[2] Since each of the 16 neuropsychological tests had a smaller interquartile range (IQR) at T2 than T1 (Supplementary Table S5), it seems likely that reduced test score variation at T2 caused the T2 data to be inadequate for PCA (which requires sufficient variation in the measures to generate any form of structure).

If aphasia recovery occurred in a unidimensional manner, one would expect the different ‘PC change’ scores to be strongly, positively correlated. However, there was no significant correlation between PC1 change and PC2 change (Spearman’s rho=-0.18, p=0.39) nor between PC2 change and PC3 change (Spearman’s rho=0.20, p=0.32), while PC1 change was significantly negatively correlated with PC3 change (Spearman’s rho=-0.62, p=0.001). These results were unchanged after partialling out T1PC1, T1PC2 and T1PC3 scores from each of the previous correlations (PC1 change vs PC2 change, Spearman’s rho=-0.47, p=0.03; PC2 change vs PC3 change, Spearman’s rho=0.19, p=0.39; PC1 change vs PC3 change, Spearman’s rho=-0.53, p=0.009).

Supplementary Figure S2: Movement through PCA space during recovery

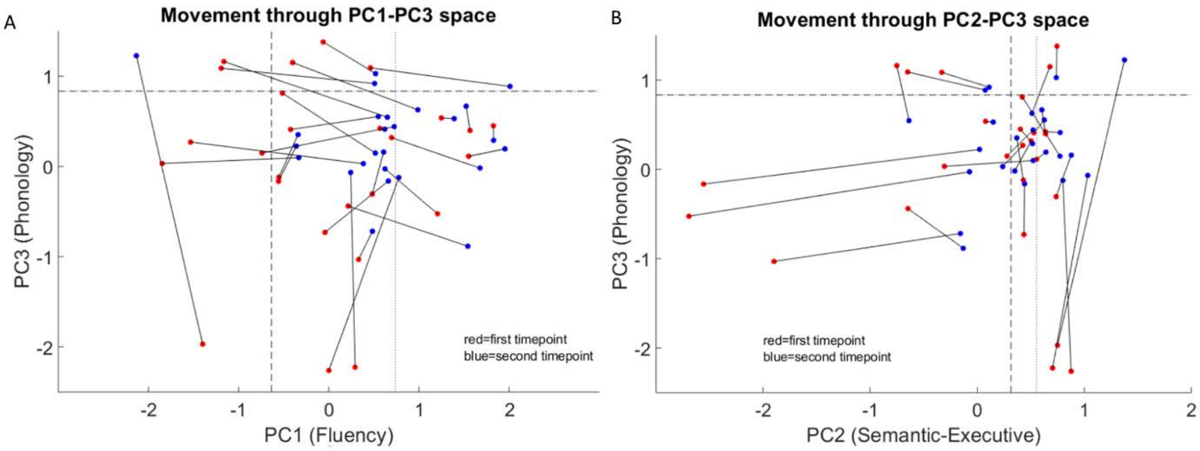

Supplementary Figure S2 Legend: Scatter plots depicting the 26 patients with post-stroke aphasia moving through Principal Component 1-Principal Component 3 space **(A)** and Principal Component 2-Principal Component 3 space **(B)** between 2 weeks and 4 months post-stroke. Each line represents an individual patient with the red circle their Timepoint 1 performance (2 weeks post-stroke) and the blue circle their Timepoint 2 performance (4 months post-stroke). Dashed lines represent the 'lower bound of normal control performance'; dotted lines represent the 'mean Principal Component score'; X and Y axis show the component scores. Abbreviations: PC = Principal Component; PC2 = 'semantic/executive' principal component; PC3 = 'phonology' principal component.

If there was a singular recovery process then the patients would move uniformly towards the upper, right-hand corner (towards control level performance). Instead, it is clear that the patients did not 'move' together through each PCA space but rather had different recovery trajectories (Fig. 2, Supplementary Figure S2). For instance, several patients improve predominantly along one PC dimension while remaining static on the other PC dimension (shown in the scatterplots as moving vertically or horizontally), while some patients are already within the limits of 'normal' at T1 and remain in a similar position of PCA space at T2.

Regional activation during speech production

A one-sample *t*-test on the 'mean activation image', averaged across T1 and T2, of 'Speech+Count>Rest' in healthy controls identified significant bilateral activation throughout frontal cortex (precentral gyrus, supplementary motor cortex, superior frontal gyrus, middle frontal gyrus [MFG], IFG pars opercularis and left IFG pars triangularis), temporal cortex (Heschl's gyrus, planum temporale, planum polare, STG, middle temporal gyrus [MTG], temporal pole), parietal cortex (postcentral gyrus, superior parietal lobule, supramarginal gyrus), insular cortex, anterior cingulate cortex, putamen and thalamus (Supplementary Figure S3A, Supplementary Table S8). This demonstrates that multiple regions throughout both hemispheres are involved in language in health. The opposite contrast, 'Rest>Speech+Count', identified significant bilateral deactivation during speech in the frontal medial cortex, subcallosal cortex, frontal pole, angular gyrus, posterior cingulate gyrus and precuneus (Supplementary Figure S3B, Supplementary Table S8).

To identify whether regional activation during speech production varied between participant groups (patients vs. controls) and timepoints (T1 vs. T2), we performed a mixed-design ANOVA with first-level 'Speech+Count>Rest' contrast estimate as the dependent variable, 'timepoint' as the within-subjects factor and 'participant group' as the between-subjects factor. The main effect of timepoint (T1 vs. T2) was not significant, meaning that regional activation during speech production, collapsed across patients and controls, did not change significantly between T1 and T2. The 'participant group*timepoint' interaction was not significant, meaning that activation change between timepoints was not significantly different in patients compared to controls. The main effect of group was assessed using an independent samples *t*-test comparing the 'mean activation images', averaged across timepoints, of patients vs. controls. We did not identify any regions of significantly greater activation in patients than controls. We found significantly less activation in patients than controls in three clusters. The first cluster encompassed right posterior cingulate and temporo-parietal cortex including the precuneus, superior parietal lobule, angular gyrus, posterior supramarginal gyrus, STG, posterior MTG, posterior inferior temporal gyrus (ITG) and temporal pole (Supplementary Figure S3C, Supplementary Table S9); this cluster overlapped with regions activated in controls in the right planum temporale, anterior STG and temporal pole, and with regions deactivated in controls in the right precuneus and angular gyrus. The second cluster was in the left precuneus, posterior cingulate gyrus, superior parietal lobule, thalamus and caudate; while the third encompassed the right thalamus and pallidum (Supplementary Figure S3C, Supplementary Table S9).

Supplementary Figure S3: Regional activation during overt speech production

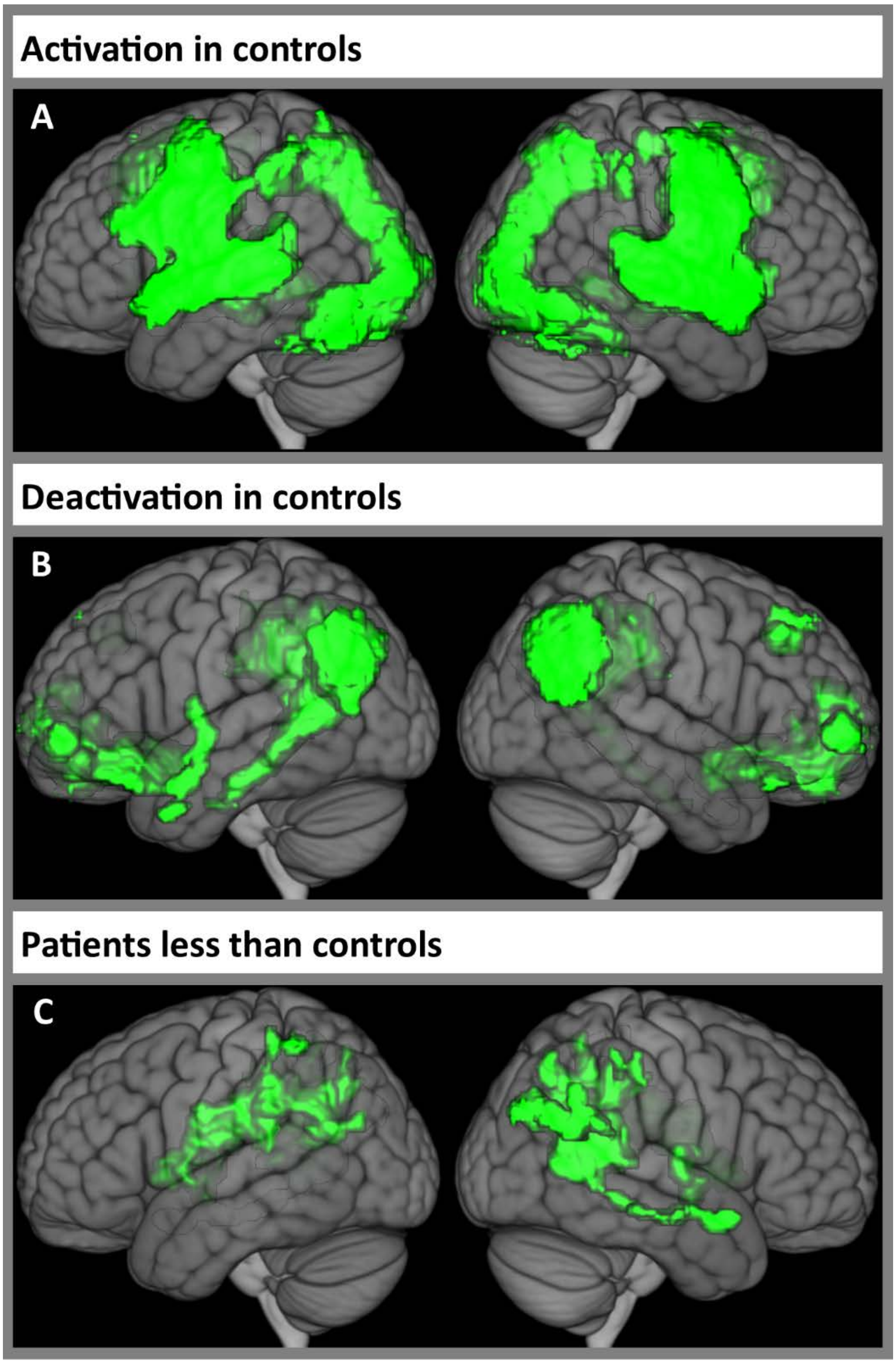

Supplementary Figure S3 Legend: **(A)** Regions of significant activation during ‘Speech+Count>Rest’, averaged across Timepoint 1 and Timepoint 2, in healthy controls (Supplementary Table S8). **(B)** Regions of significant deactivation during ‘Speech+Count>Rest’, averaged across Timepoint 1 and Timepoint 2, in healthy controls (Supplementary Table S8). **(C)** Regions of significantly less activation in patients than controls, averaged across Timepoint 1 and Timepoint 2 (Supplementary Table S9). The left and right columns show the left and right hemispheres, respectively. Statistical thresholding used a voxel-wise cluster forming threshold of $p<0.005$ (uncorrected) and a cluster-level threshold of $p<0.05$ after familywise error correction.

Supplementary Table S1: Demographic and clinical variables in participants with post-stroke aphasia

Identifier	Age	Sex	Years education	Lesion volume (cm^3)	Hours SLT	Time of first scan (days post stroke)	Time of second scan (days post stroke)	NIHSS	
								T1	T2
**Post-stroke aphasia**									
1	67	M	16	165.6	2	35	90	12	1
2	77	M	14	48.7	2	28	104	4	1
3	68	F	24	49.5	10	30	154	2	0
4	46	F	12	10.3	0	14	119	3	0
5	77	F	21	4.5	0	11	114	2	0
6	50	M	21	54.8	5	12	102	2	0
7	44	M	16	20.5	1.5	12	161	7	1
8	46	M	16	48.7	4	15	200	1	1
9	76	M	25	3.8	0	25	124	2	0
10	60	M	21	12.6	0	10	127	2	1
11	56	M	14	46.5	10	17	96	5	1
12	57	M	18	60.4	7	20	90	6	1
13	75	M	26	36.4	0	16	101	1	0
14	65	M	16	22.7	0	6	101	2	0
15	64	M	22	64.3	7	6	89	13	2
16	64	M	24	25.4	18	12	96	1	0
17	39	F	22	29.9	0	20	91	0	0
18	65	M	14	12.2	0	11	104	5	1
19	49	F	24	5.3	0	18	88	0	0
20	53	M	22	5.6	0	5	102	1	0
21	69	F	15	112.5	4	9	87	4	1
22	54	M	15	35.8	0	14	99	1	0
23	53	F	14	29.7	12	8	92	3	1
24	63	M	18	6.8	0	7	90	1	0
25	50	M	17	9.7	0	20	101	0	0
26	48	F	21	173.9	10	17	95	10	4

Abbreviations: NIHSS = National Institutes of Health Stroke Scale; SLT = Speech-Language Therapy; T1 = timepoint 1; T2 = timepoint 2.

Supplementary Table S2: Neuropsychological scores in participants with post-stroke aphasia at Timepoint 1

ID	CAT Spoken Picture Description	CAT Fluency	CAT Spoken Comprehension (/66)	CAT Written Comprehension (/62)	CAT Repetition (/74)	CAT Object Naming (/58)	CAT Reading (/70)	CAT Cognitive (/38)	Ravens (/12)	Cinderella ICW Rate (ICWs Per Second)	Cinderella Syllable Rate (Syllables Per Second)	Cinderella Total ICWs	Cinderella Narrative Aphasia Score	Decision Task IES (mean reaction time/proportion correct)	Speech Task Appropriate Minus Inappropriate ICWs	Speech Task Syllable Rate (Syllables Per Second)
Cut-off	20.7	26.1	57.9	55.0	68.8	52.8	58.8	36.7	10.5	0.02	1.76	105.6	21.5	0.50	4.94	1.69
1	42.5	6	47	47	47	48	34	24	11	0.82	2.55	87	9.3	0.55	3.00	1.02
2	60.0	12	56	50	37	44	61	33	11	0.45	1.11	210	9.8	0.47	2.33	1.04
3	38.0	10	58	56	61	55	68	33	7	0.43	1.02	41	9.1	0.57	2.75	1.36
4	69.0	25	64	61	68	57	67	37	11	0.88	2.40	220	13.6	0.41	1.87	0.62
5	108.0	36	63	61	67	57	70	38	12	1.03	2.42	129	16.9	0.50	4.98	1.21
6	34.0	7	55	48	60	43	70	33	12	0.39	1.29	75	12.3	0.47	2.18	1.17
7	0.0	0	52	33	14	6	2	31	9	0.00	0.00	0	0.0	0.32	3.78	1.00
8	44.0	10	58	47	57	45	63	33	10	0.47	1.15	131	13.2	0.39	3.05	0.82
9	61.5	15	60	57	60	57	70	38	9	0.56	1.45	112	17.0	0.40	4.58	1.89
10	40.0	33	62	59	70	57	70	38	12	0.93	2.06	245	23.4	0.41	5.05	2.04
11	31.5	5	35	27	18	14	24	27	4	0.80	1.95	72	7.7	0.63	0.67	1.21
12	22.0	2	60	46	27	26	9	32	11	0.95	2.03	95	7.5	0.37	1.13	0.91
13	115.0	37	66	62	73	57	70	36	12	1.52	3.16	280	29.1	0.38	7.18	2.40
14	31.0	11	58	55	60	47	44	32	8	0.36	0.67	20	4.0	0.35	1.40	1.07

15	26.0	4	54	48	13	20	28	35	11	0.52	1.21	104	9.7	0.42	1.74	0.80
16	106.5	11	46	27	71	47	70	33	12	0.97	1.97	170	18.8	0.37	6.45	2.61
17	73.0	35	64	62	74	56	70	38	12	1.55	3.05	277	24.0	0.39	5.78	1.90
18	52.5	23	62	53	68	54	58	33	12	0.82	2.28	14	9.6	0.40	4.18	1.37
19	87.0	52	66	62	74	58	70	38	12	1.54	3.05	250	24.4	0.36	6.12	2.23
20	122.0	26	55	61	72	58	70	37	12	1.28	2.60	231	21.6	0.45	5.40	1.90
21	63.0	4	32	15	47	23	4	36	6	0.98	1.90	159	13.4	0.89	2.20	0.95
22	25.5	9	52	44	71	44	54	30	7	0.43	0.92	73	9.9	0.49	2.43	1.83
23	11.0	0	48	34	61	35	21	34	8	0.00	0.00	0	0.0	0.51	1.58	0.55
24	62.0	22	65	60	71	57	70	38	11	0.79	1.83	81	17.2	0.37	6.43	2.50
25	68.0	31	64	61	74	58	69	38	12	1.07	2.34	91	14.2	0.33	9.28	3.17
26	-3.0	6	18	2	52	25	8	19	8	0.58	1.19	60	3.3	0.47	-3.65	1.37

Neuropsychological scores for the 26 participants with post-stroke aphasia at Timepoint 1 (2 weeks post stroke). Values indicates raw test scores except where units are provided in parentheses. Values highlighted in grey are worse than the 'aphasic cut-off', calculated as the fifth percentile of control sample data. Where available, maximum scores are indicated in parentheses. Decision Task Inverse Efficiency Score calculated as mean reaction time (in seconds) divided by the proportion of correct responses; higher Inverse Efficiency Score values indicate worse performance. See Supplementary Methods for description of how Narrative Aphasia Score and 'aphasic cut-off' were calculated. Abbreviations: CAT = Comprehensive Aphasia Test; ICW = Information Carrying Word; ID = Participant Identifier; IES = Inverse Efficiency Score.

Supplementary Table S3: Neuropsychological scores in participants with post-stroke aphasia at Timepoint 2

ID	CAT Spoken Picture Description	CAT Fluency	CAT Spoken Comprehension (/66)	CAT Written Comprehension (/62)	CAT Repetition (/74)	CAT Object Naming (/58)	CAT Reading (/70)	CAT Cognitive (/38)	Ravens (/12)	Cinderella ICW Rate (ICWs Per Second)	Cinderella Syllable Rate (Syllables Per Second)	Cinderella Total ICWs	Cinderella Narrative Aphasia Score	Decision Task IES (mean reaction time/proportion correct)	Speech Task Appropriate Minus Inappropriate ICWs	Speech Task Syllable Rate (Syllables Per Second)
Cut-off	20.7	26.1	57.9	55.0	68.8	52.8	58.8	36.7	10.5	0.02	1.76	105.6	21.5	0.50	4.94	1.69
1	81.5	12	60	56	52	52	52	36	11	1.57	3.67	172	11.9	0.59	2.08	0.84
2	71.0	23	62	59	64	50	65	38	12	0.86	2.02	216	18.4	0.53	3.20	1.18
3	63.5	24	60	56	74	58	70	38	12	1.00	2.38	167	22.5	0.49	4.93	2.35
4	66.0	57	64	62	72	57	68	37	12	0.92	2.39	179	17.4	0.39	3.58	1.17
5	108.0	36	63	61	67	57	70	38	12	1.27	2.87	324	19.5	0.49	5.22	1.66
6	86.0	30	65	60	67	58	70	37	11	0.90	2.19	249	21.6	0.43	5.47	1.88
7	58.5	28	64	58	73	56	69	37	12	0.00	0.00	0	0.0	0.32	5.65	1.61
8	56.5	16	59	54	73	57	64	32	10	0.71	1.56	143	15.2	0.37	3.43	0.69
9	61.5	23	66	61	70	58	70	38	12	0.97	2.16	227	18.9	0.43	4.40	1.47
10	40.0	25	64	61	70	58	70	38	12	1.10	2.38	256	20.9	0.36	6.08	2.35
11	74.0	12	56	48	46	39	38	34	7	0.96	2.25	174	9.7	0.41	1.82	1.15
12	62.0	27	66	60	66	52	59	37	12	0.89	2.05	145	19.2	0.34	4.33	1.42
13	115.0	37	66	62	73	57	70	36	12	1.52	3.16	280	29.1	0.36	7.08	2.10
14	64.0	16	61	54	68	52	56	37	9	1.09	2.23	189	15.2	0.38	2.27	1.36

15	96.0	31	63	60	65	51	63	38	12	1.03	2.20	191	19.8	0.37	3.97	1.42
16	236.0	34	60	61	74	58	70	38	12	1.41	2.82	271	24.7	0.36	6.48	2.64
17	73.0	50	66	62	74	58	70	38	12	1.59	3.57	300	29.3	0.34	6.38	2.27
18	74.5	28	66	59	73	58	68	37	12	1.25	2.68	129	18.3	0.39	6.02	1.91
19	87.0	52	66	62	74	58	70	38	12	1.54	3.05	250	24.4	0.35	7.59	2.73
20	122.0	43	55	61	72	58	70	37	12	1.26	2.61	243	22.1	0.43	5.03	1.90
21	69.0	20	65	50	63	46	60	38	7	0.90	1.80	240	22.1	0.51	2.99	1.01
22	64.5	17	58	56	69	48	58	32	7	1.50	3.25	90	15.5	0.46	3.53	1.75
23	52.0	15	65	53	63	55	49	34	10	0.70	1.50	108	16.2	0.41	3.45	1.08
24	58.5	41	66	62	74	58	70	38	12	1.41	3.06	160	22.2	0.43	6.08	2.26
25	76.0	35	65	62	74	58	70	38	12	1.39	2.68	151	13.8	0.33	8.93	2.95
26	51.0	10	49	43	63	52	57	36	11	0.56	1.31	87	18.5	0.44	2.48	0.89

Neuropsychological scores for the 26 participants with post-stroke aphasia at Timepoint 2 (4 months post stroke). Values indicates raw test scores except where units are provided in parentheses. Values highlighted in grey are worse than the 'aphasic cut-off', calculated as the fifth percentile of control sample data. Where available, maximum scores are indicated in parentheses. Decision Task Inverse Efficiency Score calculated as mean reaction time (in seconds) divided by the proportion of correct responses; higher Inverse Efficiency Score values indicate worse performance. See Supplementary Methods for description of how Narrative Aphasia Score and 'aphasic cut-off' were calculated. Abbreviations: CAT = Comprehensive Aphasia Test; ICW = Information Carrying Word; ID = Participant Identifier; IES = Inverse Efficiency Score.

Supplementary Table S4: Group level comparisons of neuropsychological scores between participants with post-stroke aphasia at Timepoint 1 and controls

Neuropsychological test	Patients at T1 (median, IQR)	Controls (median, IQR)	P value
CAT Fluency	11.0 (21.5)	46.0 (13.3)	<0.0005*
CAT Cognitive	33.5 (6.0)	38.0 (0.3)	<0.0005*
Ravens	11.0 (4.0)	12.0 (1.0)	0.03
Cinderella Syllables Per Second	1.93 (1.26)	3.29 (1.26)	6×10^{-7}*
Decision Task IES	0.41 (0.12)	0.35 (0.08)	0.004*
Speech Task Appropriate Minus Inappropriate ICWs	3.02 (3.66)	7.16 (1.89)	1×10^{-7}*
Speech Task Syllable Rate	1.29 (0.95)	2.69 (0.65)	<0.0005*

Results of statistical tests comparing neuropsychological scores between the post-stroke aphasia group at Timepoint 1 (n=26) and controls (n=22). 'Cinderella Syllables Per Second' and 'Speech Task Appropriate Minus Inappropriate ICWs' were normally distributed; independent samples *t*-tests were used. 'CAT Fluency', 'CAT Cognitive', 'Ravens', 'Decision Task IES', and 'Speech Task Syllable Rate' were not normally distributed; Mann-Whitney U tests were used. * indicates the p-value is significant at the Bonferroni corrected significance threshold of p<0.007 (corrected for 7 comparisons). Abbreviations: CAT = Comprehensive Aphasia Test; ICW = Information Carrying Word; IES = Inverse Efficiency Score; NAS = Narrative Aphasia Score.

Supplementary Table S5: Comparisons of neuropsychological scores between participants with post-stroke aphasia at Timepoint 1 and Timepoint 2

Neuropsychological test	Patients at T1 (median, IQR)	Patients at T2 (median, IQR)	P value
CAT Spoken Picture Description	48.3 (40.3)	70.0 (25.5)	<0.0005*
CAT Fluency	11.0 (21.5)	27.5 (19.5)	<0.0005*
CAT Spoken Comprehension	58.0 (12.3)	64.0 (6.0)	<0.0005*
CAT Written Comprehension	51.5 (19.5)	60.0 (5.8)	<0.0005*
CAT Repetition	61.0 (24.0)	70.0 (8.5)	<0.0005*
CAT Object Naming	47.5 (24.3)	57.0 (6.0)	<0.0005*
CAT Reading	65.0 (43.0)	68.5 (11.3)	<0.0005*
CAT Cognitive	33.5 (6.0)	37.0 (2.0)	0.001*
Ravens	11.0 (4.0)	12.0 (1.3)	0.002*
Cinderella ICWs Per Second	0.81 (0.55)	1.06 (0.51)	2×10^{-5}*
Cinderella Syllables Per Second	1.93 (1.26)	2.38 (0.87)	2×10^{-5}*
Cinderella Total ICWs	99.5 (143.5)	184.0 (104.8)	1×10^{-5}*
Cinderella NAS	12.7 (8.8)	19.0 (6.7)	9×10^{-6}*
Decision Task IES	0.41 (0.12)	0.40 (0.09)	0.02
Speech Task Appropriate Minus Inappropriate ICWs	3.02 (3.66)	4.67 (2.71)	0.0005*
Speech Task Syllable Rate	1.29 (0.95)	1.63 (1.10)	0.02

Results of statistical tests comparing neuropsychological scores between the post-stroke aphasia group at Timepoint 1 and Timepoint 2. 'Cinderella ICWs Per Second', 'Cinderella Syllables Per Second', 'Cinderella Total ICWs', 'Cinderella NAS' and 'Speech Task Appropriate Minus Inappropriate ICWs' were normally distributed and used paired *t*-tests. All other variables were not normally distributed and used Wilcoxon signed-rank tests. * indicates the p-value is significant at the Bonferroni corrected significance threshold of $p<0.003$ (corrected for 16 comparisons). Abbreviations: CAT = Comprehensive Aphasia Test; ICW = Information Carrying Word; IES = Inverse Efficiency Score; NAS = Narrative Aphasia Score.

Supplementary Table S6: Group level comparisons of neuropsychological scores between participants with post-stroke aphasia at Timepoint 2 and controls

Neuropsychological test	Patients at T2 (median, IQR)	Controls (median, IQR)	P value
CAT Fluency	27.5 (19.5)	46.0 (13.3)	<0.0005*
CAT Cognitive	37.0 (2.0)	38.0 (0.3)	0.01
Ravens	12.0 (1.3)	12.0 (1.0)	0.88
Cinderella Syllables Per Second	2.38 (0.87)	3.29 (1.26)	0.001*
Decision Task IES	0.40 (0.09)	0.35 (0.08)	0.04
Speech Task Appropriate Minus Inappropriate ICWs	4.67 (2.71)	7.16 (1.89)	1×10^{-6}*
Speech Task Syllable Rate	1.63 (1.10)	2.69 (0.65)	<0.0005*

Results of statistical tests comparing neuropsychological scores between the post-stroke aphasia group at Timepoint 2 (n=26) and controls (n=22). 'Cinderella Syllables Per Second' and 'Speech Task Appropriate Minus Inappropriate ICWs' were normally distributed; independent samples *t*-tests were used. 'CAT Fluency', 'CAT Cognitive', 'Ravens', 'Decision Task IES', and 'Speech Task Syllable Rate' were not normally distributed; Mann-Whitney U tests were used. * indicates the p-value is significant at the Bonferroni corrected significance threshold of p<0.007 (corrected for 7 comparisons). Abbreviations: CAT = Comprehensive Aphasia Test; ICW = Information Carrying Word; IES = Inverse Efficiency Score; NAS = Narrative Aphasia Score.

Supplementary Table S7: Estimated Principal Component scores in participants with post-stroke aphasia

Identifier	PC1		PC2		PC3	
	T1	T2	T1	T2	T1	T2
1	0.21	1.54	-0.65	-0.13	-0.44	-0.88
2	-0.04	0.66	0.44	0.44	-0.73	-0.16
3	-1.19	0.51	-0.33	0.11	1.09	0.92
4	0.48	0.61	0.74	0.88	-0.31	0.16
5	0.69	1.68	0.50	0.35	0.32	-0.02
6	-0.74	0.72	0.28	0.52	0.15	0.44
7	-1.40	-2.13	0.75	1.38	-1.97	1.22
8	-0.55	-0.34	0.43	0.37	-0.12	0.35
9	-0.51	0.52	0.42	0.77	0.81	0.15
10	0.57	0.62	0.64	0.78	0.42	0.41
11	0.33	0.48	-1.90	-0.16	-1.03	-0.72
12	0.29	0.24	0.70	1.03	-2.22	-0.07
13	1.83	1.83	0.40	0.52	0.45	0.29
14	-1.53	0.38	0.43	0.24	0.27	0.03
15	0.00	0.78	0.88	0.80	-2.26	-0.12
16	0.46	2.01	-0.65	0.08	1.09	0.89
17	1.55	1.95	0.56	0.64	0.11	0.19
18	-0.42	0.55	0.53	0.63	0.41	0.55
19	1.57	1.52	0.64	0.60	0.40	0.67
20	1.25	1.39	0.08	0.15	0.54	0.53
21	1.20	0.62	-2.69	-0.07	-0.53	-0.03
22	-1.17	0.65	-0.75	-0.64	1.16	0.55
23	-1.85	-0.33	-0.31	0.52	0.03	0.10
24	-0.40	0.99	0.68	0.51	1.15	0.63
25	-0.06	0.52	0.74	0.74	1.38	1.03
26	-0.56	-0.36	-2.56	0.02	-0.17	0.22

Estimated Principal Component scores for each of the 26 patients with post-stroke aphasia at Timepoint 1 and Timepoint 2. Neuropsychological scores at Timepoint 1 and Timepoint 2 were projected into the Timepoint 1 Principal Component Analysis space. Abbreviations: PC = Principal Component; PC1 = 'fluency' Principal Component; PC2 = 'semantic/executive' principal component; PC3 = 'phonology' principal component; T1 = Timepoint 1 (2 weeks post-stroke); T2 = Timepoint 2 (4 months post stroke).

Supplementary Table S8: Clusters of significant activation and deactivation during speech production (Supplementary Figure S3A, S3B)

Cluster	Cluster size (number of voxels)	Coordinate (x y z)	Z	Location
Activation in controls, averaged across timepoints (Supplementary Fig.S3A)				
1	44386	-44, -12, 32	7.48	L precentral gyrus
		0, 4, 60	7.36	Supplementary motor cortex
		44, -10, 34	7.35	R precentral gyrus
		62, -18, 0	6.68	R posterior superior temporal gyrus
		54, -6, 22	6.65	R precentral gyrus
		48, -6, 18	6.56	R central opercular cortex
		56, -2, 18	6.54	R precentral gyrus
		16, -16, 0	6.48	R thalamus
		64, -14, 2	6.46	R posterior superior temporal gyrus
		38, -28, 4	6.42	R Heschl's gyrus (includes H1 and H2)
		52, -4, 42	6.35	R precentral gyrus
		60, -4, 38	6.34	R precentral gyrus
		-6, 12, 58	6.26	Superior frontal gyrus
		60, -8, 0	6.21	R planum temporale
		-58, -22, 0	6.13	L posterior superior temporal gyrus
		4, 16, 42	6.12	Paracingulate gyrus
Deactivation in controls, averaged across timepoints (Supplementary Fig.S3B)				
1	2405	46, -64, 30	6.38	R superior lateral occipital cortex
		44, -72, 42	5.05	R superior lateral occipital cortex
		62, -56, 18	3.50	R angular gyrus
		64, -54, 14	3.09	R angular gyrus
2	9544	6, -52, 34	6.33	Precuneus
		8, -66, 30	6.14	Precuneus
		-6, -66, 34	5.94	Precuneus
		4, -42, 34	5.92	Posterior cingulate gyrus
		12, -56, 26	5.86	R precuneus
		-10, -60, 20	5.44	Precuneus
		-44, -70, 30	4.84	L superior lateral occipital cortex
		-42, -74, 30	4.78	L superior lateral occipital cortex
		-48, -60, 32	4.42	L angular gyrus
		-24, -42, -4	4.26	L posterior parahippocampal gyrus
		-24, -28, -20	4.23	L posterior parahippocampal gyrus
		4, -32, 46	3.89	Posterior cingulate gyrus
		-26, -46, 4	3.75	L lingual gyrus
		-32, -46, 2	3.68	L temporal occipital fusiform gyrus
		-22, -20, -26	3.51	L anterior parahippocampal gyrus
		-18, -12, -30	3.34	L anterior parahippocampal gyrus
3	6491	-2, 38, -18	5.22	Frontal medial cortex
		-4, 18, -10	4.96	Subcallosal cortex

		4, 18, -10	4.82	Subcallosal cortex
		34, 60, -2	4.78	R frontal pole
		6, 58, -6	4.65	Frontal pole
		8, 14, -14	4.63	Subcallosal cortex
		6, 10, -14	4.61	Subcallosal cortex
		4, 48, -18	4.60	Frontal medial cortex
		6, 24, -12	4.52	Subcallosal cortex
		-2, 8, -14	4.49	Subcallosal cortex
		6, 62, 2	4.42	Frontal pole
		-8, 10, -12	4.40	Subcallosal cortex
		4, 32, -16	4.37	Frontal medial cortex
		-6, 56, -6	4.35	Frontal pole
		-6, 6, -16	4.33	Subcallosal cortex
		-32, 56, -8	4.24	L frontal pole
4	340	24, 32, 38	4.42	R middle frontal gyrus
		18, 44, 46	3.20	R frontal pole
		28, 44, 46	2.72	R frontal pole
		22, 46, 48	2.66	R frontal pole
5	390	-46, 4, -22	3.84	L temporal pole
		-40, -4, -14	3.47	L insular cortex
		-52, 6, -34	3.26	L temporal pole
		-42, -4, 8	3.13	L insular cortex
		-40, -8, -2	3.04	L insular cortex
		-38, 6, -34	2.75	L temporal pole

Table showing details of local peak maxima in clusters of activation and deactivation in controls, averaged across timepoints 1 and 2. ‘Coordinate’ is the Montreal Neurological Institute coordinate of the corresponding peak. ‘Location’ of the peak coordinate is defined using the Harvard-Oxford atlas for cortical regions or the Automated Anatomical Labelling atlas for subcortical regions. Abbreviation: L = left; R = right.

Supplementary Table S9: Clusters of significantly less activation in patients than controls (Supplementary Figure S3C)

Cluster	Cluster size (number of voxels)	Coordinate (x y z)	Z	Location
Less activation in patients than controls (Supplementary Fig.S3C)				
1	3730	24, -54, 18	4.50	R precuneus
		40, -74, 32	3.88	R superior lateral occipital cortex
		46, -66, 30	3.82	R superior lateral occipital cortex
		34, -50, 16	3.71	R posterior supramarginal gyrus
		34, -40, 16	3.64	R planum temporale
		34, -42, 22	3.55	R posterior supramarginal gyrus
		36, -34, 8	3.51	R planum temporale
		20, -60, 42	3.42	R superior lateral occipital cortex
		14, -66, 46	3.41	R precuneus
		20, -64, 44	3.40	R superior lateral occipital cortex
		14, -38, 8	3.28	R posterior cingulate gyrus
		46, -28, -14	3.26	R posterior inferior temporal gyrus
		26, -44, 10	3.23	R posterior cingulate gyrus
		8, -48, 28	3.22	Posterior cingulate gyrus
		52, -54, 24	3.21	R angular gyrus
		54, -56, 36	3.20	R angular gyrus
2	1895	-18, -56, 32	4.09	L precuneus
		-22, -6, 28	3.58	L superior corona radiata
		-20, -38, 34	3.52	L posterior cingulate gyrus
		-20, -20, 20	3.47	L thalamus
		-16, -16, 16	3.45	L thalamus
		-42, -48, 58	3.45	L superior parietal lobule
		-26, -32, 26	3.44	L posterior corona radiata
		-22, -58, 24	3.41	L precuneus
		-20, -8, 22	3.40	L caudate
		-20, -46, 32	3.39	L precuneus
		-16, -4, 16	3.38	L caudate
		-26, -34, 32	3.37	L posterior corona radiata
		-12, -64, 38	3.35	L precuneus
		-22, -24, 30	3.30	L posterior corona radiata
		-10, 10, 2	3.29	L caudate
		-22, -24, 22	3.29	L caudate
3	298	12, -6, -6	3.75	R cerebral peduncle
		4, -6, 8	3.31	Thalamus
		18, -10, 4	3.29	R thalamus
		14, -6, 14	3.08	R thalamus
		18, -2, 0	3.07	R pallidum

Table showing details of local peak maxima in clusters of significantly less activation in patients with post-stroke aphasia compared to controls, averaged across Timepoints 1 and 2. This was assessed using an independent sample *t*-test comparing the 'mean activation image across timepoints' of patients vs. controls. 'Coordinate' is the Montreal Neurological Institute coordinate of the corresponding peak.

‘Location’ of the peak coordinate is defined using the Harvard-Oxford atlas for cortical regions, the Automated Anatomical Labelling atlas for subcortical regions or the John Hopkins University atlas for white matter tracts. Abbreviation: L = left; R = right.

Supplementary Table S10: Cluster in which activation was positively associated with fluency at 2 weeks post-stroke (Figure 3)

Cluster	Cluster size (number of voxels)	Coordinate (x y z)	Z	Location
1	3557	42, -36, 4	4.68	R posterior supramarginal gyrus
		60, -56, 4	4.50	R temporooccipital middle temporal gyrus
		56, -54, 6	4.46	R temporooccipital middle temporal gyrus
		42, -44, 12	4.20	R temporooccipital middle temporal gyrus
		58, -18, 20	4.02	R central opercular cortex
		42, -52, 8	3.83	R temporooccipital middle temporal gyrus
		34, -36, 38	3.73	R postcentral gyrus
		32, -42, 36	3.68	R superior parietal lobule
		52, -18, 26	3.50	R postcentral gyrus
		58, -46, 30	3.46	R angular gyrus
		46, -60, 6	3.41	R inferior lateral occipital cortex
		30, -20, 4	3.39	R insular cortex
		62, -42, 8	3.36	R temporooccipital middle temporal gyrus
		34, 2, 14	3.30	R insular cortex
		54, -68, 0	3.29	R inferior lateral occipital cortex
		64, -42, 16	3.26	R posterior supramarginal gyrus

Table showing details of local peak maxima for a cluster in which activation was significantly positively associated with Principal Component 1 'fluency' score at Timepoint 1 (2 weeks post-stroke) in patients with post-stroke aphasia. 'Coordinate' is the Montreal Neurological Institute coordinate of the corresponding peak. 'Location' of the peak coordinate is defined using the Harvard-Oxford atlas for cortical regions or the Automated Anatomical Labelling atlas for subcortical regions. Abbreviations: L = left; PC = Principal Component; R = right; T1 = Timepoint 1.

Supplementary Table S11: Regression models for cluster in which activation was positively associated with fluency at 2 weeks post-stroke (Figure 3)

Model/variable	B	SE	p-value	Adjusted R^2	N
**Model 1:<br>T1PC1 ~ 1 + mean activation**			7.1×10^{-5}*	0.47	26
Constant	0.15	0.15	0.32		
Mean activation	0.54	0.11	7.1×10^{-5}*		
**Model 2:<br>T1PC1 ~ 1 + mean activation + lesion volume + years education + age**			0.003*	0.43	26
Constant	-1.31	1.04	0.22		
Mean activation	0.51	0.12	0.0004*		
Lesion volume	0.002	0.004	0.67		
Years education	0.04	0.04	0.28		
Age	0.009	0.01	0.53		

Activation was positively associated with Principal Component 1 'fluency' score at Timepoint 1 (2 weeks post-stroke) on mass univariate analysis in one cluster. This table contains robust regression models using the mean activation extracted from this cluster to explain speech fluency at 2 weeks post-stroke in patients with post-stroke aphasia. * indicates the p-value is significant at $p<0.05$. Abbreviations: B = unstandardised regression coefficient; N=number of patients included in model; PC = Principal Component; PC1 = 'fluency' Principal Component; SE=Standard Error of regression coefficient; T1 = Timepoint 1 (2 weeks post-stroke); T2 = Timepoint 2 (4 months post stroke).

Supplementary Table S12: Regions in which increased activation was positively associated with fluency improvement between 2 weeks and 4 months post-stroke, before controlling for baseline fluency performance (Figure 4A-C)

Cluster	Cluster size (number of voxels)	Coordinate (x y z)	Z	Location
1 (Fig. 4A)	474	-46, 38, 26	6.66	L frontal pole
		-40, 52, 14	4.19	L frontal pole
		-50, 18, 42	3.58	L middle frontal gyrus
		-42, 18, 50	3.51	L middle frontal gyrus
		-50, 28, 30	3.51	L middle frontal gyrus
		-42, 26, 46	3.44	L middle frontal gyrus
		-46, 18, 46	3.39	L middle frontal gyrus
		-40, 30, 44	3.36	L middle frontal gyrus
		-48, 24, 40	3.31	L middle frontal gyrus
		-32, 60, 14	3.25	L frontal pole
		-38, 58, -2	3.14	L frontal pole
		-46, 30, 34	3.12	L middle frontal gyrus
		-40, 56, -8	3.12	L frontal pole
		-34, 46, 32	3.06	L frontal pole
		-30, 56, 20	2.99	L frontal pole
		-36, 62, -2	2.94	L frontal pole
2 (Fig. 4B)	12751	58, -60, 14	4.62	R inferior lateral occipital cortex
		64, -56, -6	4.37	R temporooccipital middle temporal gyrus
		0, -56, 0	4.23	Lingual gyrus
		-42, -64, -14	4.10	L temporal occipital fusiform cortex
		52, -74, 16	4.09	R superior lateral occipital cortex
		-36, -66, -14	4.04	L occipital fusiform gyrus
		30, -46, -28	4.01	R temporal occipital fusiform cortex
		62, -48, 6	4.01	R temporooccipital middle temporal gyrus
		-12, -38, -16	3.98	L posterior parahippocampal gyrus
		-36, -58, -10	3.98	L temporal occipital fusiform cortex
		-44, -60, -16	3.95	L temporal occipital fusiform cortex
		2, -40, -18	3.93	Vermis
		-32, -60, -22	3.91	L temporal occipital fusiform cortex
		-46, -52, -14	3.88	L temporooccipital inferior temporal gyrus
		64, -54, 12	3.86	R temporooccipital middle temporal gyrus
		56, -62, -6	3.85	R inferior lateral occipital cortex
3 (Fig. 4C)	469	50, 26, 40	3.90	R middle frontal gyrus
		38, 20, 56	3.81	R middle frontal gyrus
		50, 20, 44	3.72	R middle frontal gyrus
		52, 24, 36	3.70	R middle frontal gyrus
		56, 22, 32	3.56	R middle frontal gyrus
		46, 22, 46	3.43	R middle frontal gyrus
		42, 18, 52	3.41	R middle frontal gyrus
		44, 26, 48	3.40	R middle frontal gyrus
		30, 36, 48	3.34	R frontal pole
		32, 42, 44	3.32	R frontal pole
		54, 28, 30	3.23	R middle frontal gyrus

58, 24, 22	3.19	R inferior frontal gyrus pars triangularis
58, 20, 24	3.14	R inferior frontal gyrus pars opercularis
48, 36, 30	3.12	R frontal pole
36, 26, 54	3.11	R middle frontal gyrus
38, 42, 38	3.08	R frontal pole

Table showing details of local peak maxima for clusters in which increased activation between Timepoint 1 (2 weeks) and Timepoint 2 (4 months post-stroke) was significantly positively associated with Principal Component 1 'fluency' improvement over the same period in patients with post-stroke aphasia. 'Coordinate' is the Montreal Neurological Institute coordinate of the corresponding peak. 'Location' of the peak coordinate is defined using the Harvard-Oxford atlas for cortical regions or the Automated Anatomical Labelling atlas for subcortical regions. Abbreviations: L = left; PC = Principal Component; R = right; T1 = Timepoint 1 (2 weeks post-stroke); T2 = Timepoint 2 (4 months post-stroke).

Supplementary Table S13: Regression models for clusters in which increased activation was positively associated with fluency improvement between 2 weeks and 4 months post-stroke, before controlling for baseline fluency performance (Figure 4A-C)

Model/variable	B	SE	p-value	Adjusted R^2	N
**Cluster 1 (Fig. 4A)**					
**Model 1: PC1Change ~ 1 + mean activation change**			0.0001*	0.44	26
Constant	0.72	0.12	2.1×10^{-6}*		
Mean activation change	0.33	0.07	0.0001*		
**Model 2: PC1Change ~ 1 + mean activation change + T1PC1**			0.0002*	0.48	26
Constant	0.73	0.11	7.0×10^{-7}*		
Mean activation change	0.25	0.08	0.003*		
T1PC1	-0.22	0.12	0.09		
(No significant T1PC1*mean activation change interaction)					
**Model 3: PC1Change ~ 1 + mean activation change + T1PC1 + lesion volume + years education + age**			3.4×10^{-5}*	0.67	26
Constant	-1.06	0.61	0.10		
Mean activation change	0.29	0.07	0.0003*		
T1PC1	-0.33	0.10	0.006*		
Lesion volume	-0.003	0.002	0.11		
Years education	0.06	0.02	0.02*		
Age	0.01	0.008	0.13		
**Cluster 2 (Fig. 4B)**					
**Model 1: PC1Change ~ 1 + mean activation change**			5.6×10^{-5}*	0.48	26
Constant	0.59	0.11	1.6×10^{-5}*		
Mean activation change	0.25	0.05	5.7×10^{-5}*		
**Model 2: PC1Change ~ 1 + mean activation change + T1PC1**			0.0003*	0.46	26
Constant	0.62	0.11	9.4×10^{-6}*		
Mean activation change	0.19	0.06	0.004*		
T1PC1	-0.18	0.13	0.18		
(No significant T1PC1*mean activation change interaction)					

**Model 3:**<br>**PC1Change ~ 1 + mean activation change + T1PC1 + lesion volume + years education + age**				0.005*	0.43	26
Constant	-0.15	0.89	0.87			
Mean activation change	0.16	0.07	0.04*			
T1PC1	-0.25	0.17	0.15			
Lesion volume	-0.002	0.003	0.45			
Years education	0.008	0.03	0.82			
Age	0.01	0.01	0.28			
**Cluster 3 (Fig. 4C)**						
**Model 1:**<br>**PC1Change ~ 1 + mean activation change**				0.0003*	0.40	26
Constant	0.69	0.12	6.0×10^{-6}*			
Mean activation change	0.30	0.07	0.0003*			
**Model 2:**<br>**PC1Change ~ 1 + mean activation change + T1PC1**				0.0007*	0.42	26
Constant	0.74	0.11	1.5×10^{-6}*			
Mean activation change	0.17	0.08	0.04*			
T1PC1	-0.33	0.13	0.02*			
(No significant T1PC1*mean activation change interaction)						
**Model 3:**<br>**PC1Change ~ 1 + mean activation change + T1PC1 + lesion volume + years education + age**				3.7×10^{-5}	0.66	26
Constant	-1.18	0.62	0.07			
Mean activation change	0.30	0.07	0.0001*			
T1PC1	-0.30	0.11	0.009*			
Lesion volume	-0.005	0.002	0.04*			
Years education	0.07	0.02	0.01*			
Age	0.01	0.008	0.11			

Activation change was positively associated with Principal Component 1 'fluency' score change between Timepoint 1 (2 weeks) and Timepoint 2 (4 months post-stroke) on mass univariate analysis in three clusters. This table contains robust regression models using the mean activation change extracted from each of these three clusters to explain fluency change between 2 weeks and 4 months post-stroke in patients with post-stroke aphasia. * indicates the p-value is significant at $p<0.05$. Abbreviations: B = unstandardised regression coefficient; N=number of patients included in model; PC = Principal Component; PC1 = 'fluency' Principal Component; SE=Standard Error of regression coefficient; T1 = Timepoint 1 (2 weeks post-stroke); T2 = Timepoint 2 (4 months post stroke).

Supplementary Table S14: Regions in which increased activation was positively associated with fluency improvement between 2 weeks and 4 months post-stroke, after controlling for baseline fluency performance (Figure 4D-E)

Cluster	Cluster size (number of voxels)	Coordinate (x y z)	Z	Location
1 (Fig. 4D)	649	-6, 34, -22	4.47	Frontal medial cortex
		-2, 38, -24	4.16	Frontal medial cortex
		-8, 30, -20	4.06	Subcallosal cortex
		14, 44, -22	3.92	R frontal pole
		0, 38, -30	3.78	Frontal medial cortex
		-12, 10, -26	3.73	L frontal orbital cortex
		6, 36, -26	3.72	Frontal medial cortex
		16, 50, -24	3.62	R frontal pole
		-18, 28, -24	3.57	L frontal orbital cortex
		-4, 12, -26	3.57	Subcallosal cortex
		-8, 14, -26	3.57	Subcallosal cortex
		-16, 32, -24	3.57	L frontal orbital cortex
		-12, 28, -20	3.57	L frontal orbital cortex
		-20, 34, -26	3.55	L frontal orbital cortex
		20, 38, -24	3.54	R frontal pole
		-18, 56, -20	3.47	L frontal pole
2 (Fig. 4E)	735	52, -74, 16	4.34	R superior lateral occipital cortex
		58, -60, 14	4.26	R inferior lateral occipital cortex
		56, -68, 14	3.90	R inferior lateral occipital cortex
		68, -36, 32	3.72	R posterior supramarginal gyrus
		64, -54, 14	3.52	R angular gyrus
		64, -56, -6	3.50	R temporooccipital middle temporal gyrus
		60, -60, 22	3.46	R angular gyrus
		62, -62, 2	3.38	R inferior lateral occipital cortex
		64, -50, 18	3.30	R angular gyrus
		60, -62, -2	3.27	R inferior lateral occipital cortex
		68, -44, 12	3.22	R posterior supramarginal gyrus
		66, -46, 26	3.15	R angular gyrus
		60, -50, 12	3.08	R temporooccipital middle temporal gyrus
		68, -42, 20	3.00	R posterior supramarginal gyrus
		62, -48, 6	2.95	R posterior supramarginal gyrus
		58, -66, -2	2.93	R inferior lateral occipital cortex

Table showing details of local peak maxima for clusters in which increased activation between Timepoint 1 (2 weeks) and Timepoint 2 (4 months post-stroke) was significantly positively associated with Principal Component 1 'fluency' improvement, controlling for baseline Principal Component 1 score at 2 weeks, in patients with post-stroke aphasia. 'Coordinate' is the Montreal Neurological Institute coordinate of the corresponding peak. 'Location' of the peak coordinate is defined using the Harvard-Oxford atlas for cortical regions or the Automated Anatomical Labelling atlas for subcortical regions. Abbreviations: L = left; PC = Principal Component; PC1 = 'fluency' Principal Component; R = right; T1 = Timepoint 1 (2 weeks post-stroke); T2 = Timepoint 2 (4 months post-stroke).

Supplementary Table S15: Regression models for clusters in which increased activation was positively associated with fluency improvement between 2 weeks and 4 months post-stroke, after controlling for baseline fluency performance (Figure 4D-E)

Model/variable	B	SE	p-value	Adjusted R^2	N
**Cluster 1 (Fig. 4D)**					
**Model 1:**<br>**PC1Change ~ 1 + mean activation change**			0.02*	0.19	26
Constant	0.72	0.14	$3.3x10^{-5}$*		
Mean activation change	0.32	0.12	0.02*		
**Model 2:**<br>**PC1Change ~ 1 + mean activation change + T1PC1**			$1.1x10^{-5}$	0.60	26
Constant	0.71	0.10	$1.8x10^{-7}$*		
Mean activation change	0.41	0.09	$8.8x10^{-5}$*		
T1PC1	-0.49	0.10	$5.3x10^{-5}$*		
(No significant T1PC1*mean activation change interaction)					
**Model 3:**<br>**PC1Change ~ 1 + mean activation change + T1PC1 + lesion volume + years education + age**			$3.5x10^{-5}$*	0.67	26
Constant	-0.48	0.62	0.45		
Mean activation change	0.38	0.08	0.0001*		
T1PC1	-0.57	0.10	$9.5x10^{-6}$*		
Lesion volume	-0.002	0.002	0.33		
Years education	0.04	0.02	0.08		
Age	0.007	0.009	0.40		
**Cluster 2 (Fig. 4E)**					
**Model 1:**<br>**PC1Change ~ 1 + mean activation change**			$3.3x10^{-5}$*	0.50	26
Constant	0.68	0.11	$1.8x10^{-6}$*		
Mean activation change	0.31	0.06	$3.4x10^{-5}$*		
**Model 2:**<br>**PC1Change ~ 1 + mean activation change + T1PC1**			$4.4x10^{-5}$*	0.55	26
Constant	0.69	0.10	$7.4x10^{-7}$*		
Mean activation change	0.27	0.06	0.0004*		
T1PC1	-0.20	0.11	0.09		
(No significant T1PC1*mean activation change interaction)					

**Model 3: PC1Change ~ 1 + mean activation change + T1PC1 + lesion volume + years education + age**			0.0007*	0.54	26
Constant	-0.31	0.77	0.69		
Mean activation change	0.23	0.07	0.003*		
T1PC1	-0.28	0.13	0.04*		
Lesion volume	-0.002	0.002	0.44		
Years education	0.03	0.03	0.38		
Age	0.01	0.01	0.33		

Activation change was positively associated with Principal Component 1 'fluency' score change, after controlling for baseline 'fluency' score, between Timepoint 1 (2 weeks) and Timepoint 2 (4 months post-stroke) on mass univariate analysis in two clusters. This table contains robust regression models using the mean activation change extracted from each of these two clusters to explain fluency change between 2 weeks and 4 months post-stroke in patients with post-stroke aphasia. * indicates the p-value is significant at $p<0.05$. Abbreviations: B = unstandardised regression coefficient; N=number of patients included in model; PC = Principal Component; PC1 = 'fluency' Principal Component; SE=Standard Error of regression coefficient; T1 = Timepoint 1.

Supplementary Table S16: Regions in which increased activation was negatively associated with semantic/executive improvement between 2 weeks and 4 months post-stroke, after controlling for baseline semantic/executive score (Figure 5)

Cluster	Cluster size (number of voxels)	Coordinate (x y z)	Z	Location
1 (Fig. 5A)	1110	-38, 16, -42	4.88	L temporal pole
		18, 40, -20	4.54	R frontal pole
		-40, 18, -38	4.51	L temporal pole
		2, 40, -24	4.36	Frontal medial cortex
		-18, 52, -20	4.35	L frontal pole
		-34, 8, -46	4.31	L temporal pole
		-44, 14, -38	4.24	L temporal pole
		-18, 46, -22	4.22	L frontal pole
		-16, 42, -18	4.17	L frontal pole
		-42, 18, -34	4.14	L temporal pole
		-30, 20, -38	4.08	L temporal pole
		-10, 46, -22	4.04	L frontal pole
		22, 40, -22	4.02	R frontal pole
		-6, 40, -20	4.01	Frontal medial cortex
		-10, 42, -20	4.00	L frontal pole
		-18, 36, -20	3.95	L frontal pole
2 (Fig. 5B)	448	44, -14, -40	4.70	R posterior inferior temporal gyrus
		56, -4, -40	4.46	R anterior inferior temporal gyrus
		64, -26, -24	4.27	R posterior inferior temporal gyrus
		56, -32, -24	4.27	R posterior inferior temporal gyrus
		48, -22, -28	4.26	R posterior inferior temporal gyrus
		58, -28, -26	4.26	R posterior inferior temporal gyrus
		58, -24, -28	4.25	R posterior inferior temporal gyrus
		52, -24, -26	4.24	R posterior inferior temporal gyrus
		64, -22, -28	4.18	R posterior inferior temporal gyrus
		56, -10, -40	4.04	R posterior inferior temporal gyrus
		46, -20, -32	3.83	R posterior inferior temporal gyrus
		50, -14, -36	3.59	R posterior inferior temporal gyrus
		56, -2, -36	3.31	R anterior middle temporal gyrus
		56, -40, -22	3.26	R temporooccipital inferior temporal gyrus
		58, -6, -36	3.14	R temporal pole
		58, -12, -34	3.08	R posterior inferior temporal gyrus

Table showing details of local peak maxima for clusters in which increased activation between Timepoint 1 (2 weeks) and Timepoint 2 (4 months post-stroke) was significantly negatively associated with Principal Component 2 'semantic/executive' improvement, controlling for Principal Component 2 score at 2 weeks, in patients with post-stroke aphasia. 'Coordinate' is the Montreal Neurological Institute coordinate of the corresponding peak. 'Location' of the peak coordinate is defined using the Harvard-Oxford atlas for cortical regions or the Automated Anatomical Labelling atlas for subcortical regions. Abbreviations: L = left; PC = Principal Component; R = right; T1 = Timepoint 1 (2 weeks post-stroke); T2 = Timepoint 2 (4 months post-stroke).

Supplementary Table S17: Regression models for clusters in which increased activation was negatively associated with semantic/executive improvement between 2 weeks and 4 months post-stroke, after controlling for baseline semantic/executive score (Figure 5)

Model/variable	B	SE	p-value	Adjusted R^2	N
**Cluster 1 (Fig. 5A)**					
**Model 1:<br>PC2Change ~ 1 + mean activation change**			0.003*	0.28	26
Constant	0.16	0.08	0.06		
Mean activation change	-0.01	0.07	0.84		
**Model 2:<br>PC2Change ~ 1 + mean activation change + T1PC2**			2.9×10^{-15}*	0.94	26
Constant	0.39	0.04	1.8×10^{-10}*		
Mean activation change	-0.22	0.03	2.5×10^{-7}*		
T1PC2	-0.75	0.04	6.2×10^{-16}*		
(No significant T1PC2*mean activation change interaction)					
**Model 3:<br>PC2Change ~ 1 + mean activation change + T1PC2 + lesion volume + years education + age**			4.1×10^{-12}*	0.94	26
Constant	0.77	0.26	0.008*		
Mean activation change	-0.22	0.03	3.0×10^{-6}*		
T1PC2	-0.77	0.06	1.0×10^{-11}*		
Lesion volume	-0.0005	0.001	0.66		
Years education	-0.008	0.01	0.43		
Age	-0.003	0.004	0.38		
**Cluster 2 (Fig. 5B)**					
**Model 1:<br>PC2Change ~ 1 + mean activation change**			0.004*	0.27	26
Constant	0.17	0.08	0.05*		
Mean activation change	-0.03	0.10	0.79		
**Model 2:<br>PC2Change ~ 1 + mean activation change + T1PC2**			6.2×10^{-13}*	0.91	26
Constant	0.47	0.05	7.1×10^{-10}*		
Mean activation change	-0.28	0.06	8.0×10^{-5}*		
T1PC2	-0.74	0.05	1.3×10^{-13}*		
(No significant T1PC2*mean activation change interaction)					

**Model 3:** **PC2Change ~ 1 + mean activation change + T1PC2 + lesion volume + years education + age**			6.2×10^{-11}*	0.91	26
Constant	1.00	0.30	0.003*		
Mean activation change	-0.29	0.06	5.2×10^{-5}*		
T1PC2	-0.71	0.06	2.9×10^{-10}*		
Lesion volume	0.0009	0.001	0.49		
Years education	-0.007	0.01	0.52		
Age	-0.007	0.004	0.10		

Activation change was negatively associated with Principal Component 2 'semantic/executive' score change, after controlling for baseline 'semantic/executive' score, between Timepoint 1 (2 weeks) and Timepoint 2 (4 months post-stroke) on mass univariate analysis in two clusters. This table contains robust regression models using the mean activation change extracted from each of these two clusters to explain semantic/executive change between 2 weeks and 4 months post-stroke in patients with post-stroke aphasia. * indicates the p-value is significant at $p<0.05$. Abbreviations: B = unstandardised regression coefficient; N=number of patients included in model; PC = Principal Component; PC2 = 'semantic/executive' principal component; SE=Standard Error of regression coefficient; T1 = Timepoint 1.

Supplementary Table S18: Regions in which increased activation was negatively associated with phonology improvement between 2 weeks and 4 months post-stroke, before controlling for baseline phonology score (Figure 6A-C)

Cluster	Cluster size (number of voxels)	Coordinate (x y z)	Z	Location
1 (Fig. 6A)	2056	36, 4, 62	5.41	R middle frontal gyrus
		48, 8, 48	4.19	R middle frontal gyrus
		-44, 0, 46	3.91	L precentral gyrus
		4, 38, 50	3.90	Superior frontal gyrus
		52, -28, 52	3.84	R postcentral gyrus
		-38, 2, 56	3.82	L middle frontal gyrus
		-44, 2, 50	3.72	L middle frontal gyrus
		2, 32, 52	3.71	Superior frontal gyrus
		52, -8, 52	3.64	R precentral gyrus
		30, 16, 58	3.63	R middle frontal gyrus
		0, 2, 60	3.59	Supplementary motor cortex
		-30, 8, 60	3.51	L middle frontal gyrus
		12, 34, 52	3.51	R superior frontal gyrus
		-34, 8, 58	3.51	L middle frontal gyrus
		-2, 8, 66	3.50	Supplementary motor cortex
		0, 4, 64	3.48	Supplementary motor cortex
2 (Fig. 6B)	1832	-6, 38, -22	4.28	Frontal medial cortex
		-2, 38, -24	4.23	Frontal medial cortex
		-18, 52, -20	4.23	L frontal pole
		12, 44, -24	4.07	R frontal pole
		-18, 44, -24	4.04	L frontal pole
		-14, 64, -10	3.98	L frontal pole
		-16, 48, -22	3.90	L frontal pole
		-18, 28, -22	3.85	L frontal orbital cortex
		14, 48, -24	3.85	R frontal pole
		-16, 24, -22	3.81	L frontal orbital cortex
		14, 64, -4	3.77	R frontal pole
		20, 42, -24	3.75	R frontal pole
		-20, 48, -16	3.74	L frontal pole
		-8, 32, -20	3.71	Frontal medial cortex
		2, 36, -32	3.64	Frontal medial cortex
		4, 40, -32	3.64	Frontal medial cortex
3 (Fig. 6C)	819	-40, 56, -2	3.79	L frontal pole
		-48, 38, 22	3.64	L frontal pole
		-24, 44, 28	3.60	L frontal pole
		-42, 52, 6	3.51	L frontal pole
		-40, 44, 20	3.47	L frontal pole
		-32, 58, 0	3.42	L frontal pole
		-40, 56, -8	3.41	L frontal pole
		-40, 54, -14	3.38	L frontal pole
		-46, 38, 26	3.25	L frontal pole
		-20, 54, 18	3.12	L frontal pole
		-28, 40, 38	3.12	L frontal pole

	-36, 52, -16	3.10	L frontal pole
	-34, 44, 8	3.06	L frontal pole
	-48, 38, 10	2.80	L frontal pole
	-40, 32, 30	2.76	L middle frontal gyrus
	-38, 36, 28	2.75	L middle frontal gyrus

Table showing details of local peak maxima for clusters in which increased activation between Timepoint 1 (2 weeks) and Timepoint 2 (4 months post-stroke) was significantly negatively associated with Principal Component 3 'phonology' improvement, controlling for Principal Component 3 score at 2 weeks, in patients with post-stroke aphasia. 'Coordinate' is the Montreal Neurological Institute coordinate of the corresponding peak. 'Location' of the peak coordinate is defined using the Harvard-Oxford atlas for cortical regions or the Automated Anatomical Labelling atlas for subcortical regions. Abbreviations: L = left; PC = Principal Component; R = right; T1 = Timepoint 1 (2 weeks post-stroke); T2 = Timepoint 2 (4 months post-stroke).

Supplementary Table S19: Regression models for clusters in which increased activation was negatively associated with phonology improvement between 2 weeks and 4 months post-stroke, before controlling for baseline phonology score (Figure 6A-C)

Model/variable	B	SE	p-value	Adjusted R^2	N
**Cluster 1 (Fig. 6A)**					
**Model 1:** **PC3Change ~ 1 + mean activation change**			$6.4x10^{-5}$*	0.47	26
Constant	0.08	0.11	0.44		
Mean activation change	-0.27	0.06	$8.3x10^{-5}$*		
**Model 2:** **PC3Change ~ 1 + mean activation change + T1PC3 + T1PC3*mean activation change**			$1.3x10^{-12}$*	0.92	26
Constant	0.17	0.08	0.04*		
Mean activation change	-0.20	0.07	0.006*		
T1PC3	-0.49	0.10	$6.9x10^{-5}$*		
T1PC3*mean activation change	0.14	0.04	0.0009*		
**Model 3:** **PC3Change ~ 1 + mean activation change + T1PC3 + T1PC3*mean activation change + lesion volume + years education + age**			$3.7x10^{-10}$*	0.91	26
Constant	0.84	0.50	0.11		
Mean activation change	-0.18	0.07	0.02*		
T1PC3	-0.50	0.13	0.0008*		
T1PC3*mean activation change	0.13	0.04	0.002*		
Lesion volume	-0.0005	0.002	0.79		
Years education	0.002	0.02	0.94		
Age	-0.01	0.007	0.11		
**Cluster 2 (Fig. 6B)**					
**Model 1:** **PC3Change ~ 1 + mean activation change**			0.005*	0.26	26
Constant	0.10	0.12	0.39		
Mean activation change	-0.19	0.07	0.008*		
**Model 2:** **PC3Change ~ 1 + mean activation change + T1PC3**			$2.7x10^{-9}$*	0.81	26
Constant	0.23	0.07	0.002*		
Mean activation change	-0.08	0.04	0.09		
T1PC3	-0.60	0.08	$1.2x10^{-7}$*		

**Cluster 3 (Fig. 6C)**					
**Model 1:** **PC3Change ~ 1 + mean activation change**			0.003*	0.28	26
Constant	0.08	0.12	0.49		
Mean activation change	-0.18	0.06	0.006*		
**Model 2:** **PC3Change ~ 1 + mean activation change + T1PC3**			3.4×10^{-9}*	0.80	26
Constant	0.21	0.07	0.005*		
Mean activation change	-0.10	0.04	0.02*		
T1PC3	-0.57	0.08	2.1×10^{-7}*		
(No significant T1PC3*mean activation change interaction)					
**Model 3:** **PC3Change ~ 1 + mean activation change + T1PC3 + lesion volume + years education + age**			1.7×10^{-7}*	0.81	26
Constant	0.88	0.51	0.10		
Mean activation change	-0.11	0.06	0.08		
T1PC3	-0.57	0.12	8.1×10^{-5}*		
Lesion volume	-0.0004	0.002	0.84		
Years education	0.0001	0.02	0.99		
Age	-0.01	0.006	0.09		

Activation change was negatively associated with Principal Component 3 'phonology' score change between Timepoint 1 (2 weeks) and Timepoint 2 (4 months post-stroke) on mass univariate analysis in three clusters. This table contains robust regression models using the mean activation change extracted from each of these three clusters to explain phonology change between 2 weeks and 4 months post-stroke in patients with post-stroke aphasia. * indicates the p-value is significant at $p<0.05$. Abbreviations: B = unstandardised regression coefficient; N=number of patients included in model; PC = Principal Component; PC3 = 'phonology' principal component; SE=Standard Error of regression coefficient; T1 = Timepoint 1 (2 weeks post-stroke); T2 = Timepoint 2 (4 months post stroke).

Supplementary Table S20: Regions in which increased activation was negatively associated with phonology improvement between 2 weeks and 4 months post-stroke, after controlling for baseline phonology performance (Figure 6D-F)

Cluster	Cluster size (number of voxels)	Coordinate (x y z)	Z	Location
1 (Fig. 6D)	4088	14, 58, 36	5.59	R frontal pole
		12, 64, 26	5.31	R frontal pole
		14, 62, 32	4.85	R frontal pole
		22, 54, 38	4.62	R frontal pole
		20, 62, 24	4.59	R frontal pole
		26, 48, 42	4.33	R frontal pole
		28, 56, 28	4.21	R frontal pole
		8, 56, 28	4.15	Frontal pole
		-22, 60, 26	4.11	L frontal pole
		14, 54, 42	4.09	R frontal pole
		-16, 46, 36	4.05	L frontal pole
		16, 46, 38	4.04	R frontal pole
		2, 46, 42	4.03	Superior frontal gyrus
		-2, 56, 28	3.99	Superior frontal gyrus
		18, 42, 42	3.94	R frontal pole
		-10, 56, 36	3.86	L frontal pole
2 (Fig. 6E)	435	-10, 18, -20	4.27	Subcallosal cortex
		-16, 36, -22	4.20	L frontal pole
		-20, 30, -24	3.97	L frontal orbital cortex
		-16, 24, -22	3.92	L frontal orbital cortex
		0, 38, -26	3.92	Frontal medial cortex
		-8, 28, -18	3.91	Subcallosal cortex
		-6, 30, -22	3.90	Subcallosal cortex
		-12, 34, -20	3.85	L frontal orbital cortex
		20, 38, -24	3.83	R frontal pole
		6, 36, -26	3.82	Frontal medial cortex
		-6, 16, -26	3.78	Subcallosal cortex
		4, 38, -32	3.78	Frontal medial cortex
		-4, 12, -26	3.78	Subcallosal cortex
		-14, 30, -20	3.78	L frontal orbital cortex
		-4, 40, -24	3.73	Frontal medial cortex
		8, 44, -26	3.34	Frontal pole
3 (Fig. 6F)	2868	-6, -70, 56	3.98	Precuneus
		-4, -66, 42	3.89	Precuneus
		2, -70, 42	3.74	Precuneus
		8, -64, 64	3.63	Superior lateral occipital cortex
		4, -64, 48	3.62	Precuneus
		0, -88, 32	3.62	Cuneus
		8, -64, 44	3.59	Precuneus
		4, -62, 56	3.51	Precuneus
		4, -86, 36	3.50	Cuneus
		-4, -96, 24	3.49	Occipital pole
		-2, -62, 58	3.49	Precuneus

-18, -70, 48	3.36	L superior lateral occipital cortex
-4, -80, 46	3.36	Precuneus
20, -98, 10	3.32	R occipital pole
-8, -80, 48	3.30	Superior lateral occipital cortex
20, -70, 42	3.27	R superior lateral occipital cortex

Table showing details of local peak maxima for clusters in which increased activation between Timepoint 1 (2 weeks) and Timepoint 2 (4 months post-stroke) was significantly negatively associated with Principal Component 3 'phonology' improvement, controlling for Principal Component 3 score at 2 weeks, in patients with post-stroke aphasia. 'Coordinate' is the Montreal Neurological Institute coordinate of the corresponding peak. 'Location' of the peak coordinate is defined using the Harvard-Oxford atlas for cortical regions or the Automated Anatomical Labelling atlas for subcortical regions. Abbreviations: L = left; PC = Principal Component; R = right; T1 = Timepoint 1 (2 weeks post-stroke); T2 = Timepoint 2 (4 months post-stroke).

Supplementary Table S21: Regression models for clusters in which increased activation was negatively associated with phonology improvement between 2 weeks and 4 months post-stroke, after controlling for baseline phonology score (Figure 6D-F)

Model/variable	B	SE	p-value	Adjusted R^2	N
**Cluster 1 (Fig. 6D)**					
**Model 1: PC3Change ~ 1 + mean activation change**			0.03*	0.15	26
Constant	0.04	0.11	0.74		
Mean activation change	-0.05	0.06	0.50		
**Model 2: PC3Change ~ 1 + mean activation change + T1PC3 + T1PC3*mean activation change**			1.3×10^{-11}*	0.90	26
Constant	0.24	0.05	0.0001*		
Mean activation change	-0.13	0.03	0.0006*		
T1PC3	-0.65	0.06	3.7×10^{-10}*		
T1PC3*mean activation change	0.08	0.04	0.05*		
**Model 3: PC3Change ~ 1 + mean activation change + T1PC3 + T1PC3*mean activation change + lesion volume + years education + age**			1.5×10^{-9}*	0.90	26
Constant	0.68	0.40	0.10		
Mean activation change	-0.14	0.05	0.006*		
T1PC3	-0.64	0.08	1.5×10^{-7}*		
T1PC3*mean activation change	0.13	0.04	0.01*		
Lesion volume	0.001	0.002	0.54		
Years education	-0.0005	0.02	0.98		
Age	-0.008	0.006	0.19		
**Cluster 2 (Fig. 6E)**					
**Model 1: PC3Change ~ 1 + mean activation change**			1.3×10^{-6}*	0.61	26
Constant	0.04	0.10	0.66		
Mean activation change	-0.59	0.09	1.9×10^{-6}*		
**Model 2: PC3Change ~ 1 + mean activation change + T1PC3**			5.4×10^{-12}	0.89	26
Constant	0.24	0.06	0.0006*		
Mean activation change	-0.32	0.06	4.2×10^{-5}*		
T1PC3	-0.68	0.07	4.6×10^{-10}*		

(No significant T1PC3*mean activation change interaction)					
**Model 3: PC3Change ~ 1 + mean activation change + T1PC3 + lesion volume + years education + age**			$1.1x10^{-10}$*	0.91	26
Constant	0.47	0.38	0.24		
Mean activation change	-0.28	0.06	0.0001*		
T1PC3	-0.75	0.07	$3.2x10^{-10}$*		
Lesion volume	-0.003	0.001	0.04*		
Years education	0.03	0.01	0.09		
Age	-0.01	0.005	0.08		
**Cluster 3 (Fig. 6F)**					
**Model 1: PC3Change ~ 1 + mean activation change**			0.02*	0.17	26
Constant	0.009	0.11	0.94		
Mean activation change	-0.03	0.06	0.62		
**Model 2: PC3Change ~ 1 + mean activation change + T1PC3 + T1PC3*mean activation change**			$1.2x10^{-10}$*	0.87	26
Constant	0.27	0.06	0.0004*		
Mean activation change	-0.15	0.03	0.0002*		
T1PC3	-0.74	0.07	$8.4x10^{-10}$*		
T1PC3*mean activation change	0.09	0.04	0.03*		
**Model 3: PC3Change ~ 1 + mean activation change + T1PC3 + T1PC3*mean activation change + lesion volume + years education + age**			$9.2x10^{-9}$*	0.88	26
Constant	0.70	0.44	0.13		
Mean activation change	-0.12	0.04	0.007*		
T1PC3	-0.77	0.08	$1.4x10^{-8}$*		
T1PC3*mean activation change	0.07	0.04	0.09		
Lesion volume	-0.001	0.002	0.52		
Years education	0.01	0.02	0.53		
Age	-0.01	0.006	0.13		

Activation change was negatively associated with Principal Component 3 'phonology' score change, after controlling for baseline 'phonology' score, between Timepoint 1 (2 weeks) and Timepoint 2 (4 months post-stroke) on mass univariate analysis in three clusters. This table contains robust regression models using the mean activation change extracted from each of these three clusters to explain phonology change between 2 weeks and 4 months post-stroke in patients with post-stroke aphasia. * indicates the p-value is significant at $p<0.05$. Abbreviations: B = unstandardised regression coefficient; N=number of patients included in model; PC = Principal Component; PC3 = 'phonology' principal component; SE=Standard Error of regression coefficient; T1 = Timepoint 1.

References

1. Cattell R. The scree test for the number of factors. *Multivariate Behavioral Research*. 1966;1:245-276.
2. Kaiser H. An index of factor simplicity. *Psychometrika*. 1974;39:31-36.
